# Host gene constraints and genomic context impact the expression and evolution of human microRNAs

**DOI:** 10.1038/ncomms11438

**Published:** 2016-04-25

**Authors:** Gustavo S. França, Maria D. Vibranovski, Pedro A. F. Galante

**Affiliations:** 1 Centro de Oncologia Molecular, Hospital Sírio-Libanês, Rua Daher Cutait 69, 01308-060 São Paulo, Brazil; 2 Departamento de Bioquímica, Instituto de Química, Universidade de São Paulo, Av. Prof. Lineu Prestes 748, 05508-000 São Paulo, Brazil; 3 Departamento de Genética e Biologia Evolutiva, Universidade de São Paulo, Rua do Matao 277, 05508-090 São Paulo, Brazil

## Abstract

Increasing evidence has shown that recent miRNAs tend to emerge within coding genes. Here we conjecture that human miRNA evolution is tightly influenced by the genomic context, especially by host genes. Our findings show a preferential emergence of intragenic miRNAs within old genes. We found that miRNAs within old host genes are significantly more broadly expressed than those within young ones. Young miRNAs within old genes are more broadly expressed than their intergenic counterparts, suggesting that young miRNAs have an initial advantage by residing in old genes, and benefit from their hosts' expression control and from the exposure to diverse cellular contexts and target genes. Our results demonstrate that host genes may provide stronger expression constraints to intragenic miRNAs in the long run. We also report associated functional implications, highlighting the genomic context and host genes as driving factors for the expression and evolution of human miRNAs.

## Abstract (web summary)

Recent miRNAs tend to emerge within coding genes. Here, by analysing miRNA expression data from six species and comparing genomes from 13 species, the authors report that host genes may provide stronger expression constraints to intragenic miRNAs in the long run.

## Graphical abstract

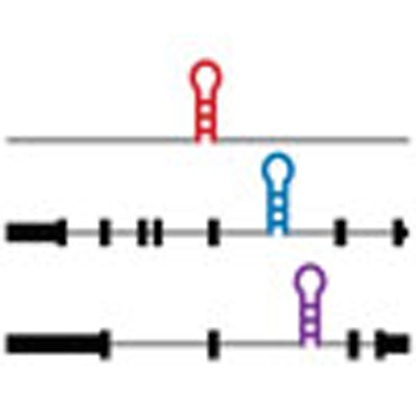

More than 20 years after microRNA (miRNA) discovery, followed by extensive research on its molecular characterization, we are currently aware of the broad impact of these small non-coding RNAs on the post-transcriptional regulation of gene expression. They are usually derived from a longer primary hairpin-shaped RNA which is cleaved by Drosha and Dicer, releasing a ∼60–80-nt precursor molecule (pre-miRNA) and a ∼21–24-nt mature miRNA, respectively^1^. Mature miRNAs typically repress mRNA expression by either translational inhibition or mRNA degradation through a perfect pairing of the `seed region' with binding sites located in the 3′ untranslated region (UTR) of target mRNAs^2^. MiRNAs participate in almost all cellular processes, several pathological conditions^3^ and in the rise of evolutionary innovations^4^.

Although many animal miRNAs are deeply conserved^5^,^6^, unlike protein-coding genes, miRNA evolution is clearly a quite dynamic process, characterized by high birth and death rates and lineage-specific expansions^7^,^8^,^9^. These expansions have been involved in development of species-specific phenotypes^10^ and establishment of morphological complexity in vertebrates^11^. Among mechanisms leading to the origin of new miRNAs such as local and non-local duplications, *de novo* origin on inter- or intragenic regions, transposable elements and other RNAs^8^, duplication and *de novo* emergence had a major contribution to the miRNA repertoire in mammals^9^. Since unstructured hairpins are commonly found across genomes, *de novo* origin of miRNAs requires specific mutations allowing hairpin recognition by the miRNA maturation machinery and a transcriptionally active environment^12^. Once new miRNAs emerge, they are typically tissue-specific and weakly expressed^9^,^12^, which can later on persist or disappear quickly^9^,^13^. After an initial adaptive evolution, preserved miRNAs can shift to a conservative phase, being gradually expressed at higher levels and in a broader range of tissues, more effectively integrated into transcriptional networks and switch to slower evolutionary rates^13^.

A notable observation is the high prevalence (>50%) of vertebrate miRNAs emerging within coding genes^9^,^14^,^15^,^16^,^17^, mostly (>80%) in the host gene sense strand^9^,^15^,^16^,^17^. MiRNAs in intronic regions were shown to be highly overrepresented, especially for those that emerged after the bird–mammal split^9^. Examples of coordinated expression of intragenic miRNAs and their host genes have been reported^14^,^18^, as well as functional relationships involving regulation of their own hosts^19^ or genes acting on related pathways^15^,^20^. Given the functional importance and a suggestive selective advantage favouring such genomic organization, here we conjectured that the evolutionary context in which miRNAs emerge may be decisive to their expression and therefore evolution. It is well-recognized that old genes, compared with young ones, tend to evolve slowly, are more broadly expressed and subjected to strong purifying selection^21^,^22^,^23^. Thus, according to our hypothesis, the age of host genes would exert strong influence on intragenic miRNAs and their evolutionary fate. Depending on their genomic context, more likely ‘proto'^24^ or young miRNAs are to persist.

Here we assessed the evolutionary impact of genomic positioning of human miRNAs by analysing their age, and the age of their host genes in the case of intragenic miRNAs. We observed a substantial increase of intragenic miRNAs in primates and a biased emergence within old host genes. We demonstrate that host gene age indeed affect the expression breadth of intragenic miRNAs. Specifically, miRNAs within young genes tend to be more tissue-specific, while young miRNAs within old genes are more broadly expressed than young intergenic ones. By comparing miRNA expression between species, we found that older intragenic miRNAs have lower expression divergence compared with their intergenic counterparts. We therefore propose that old host genes offer a suitable environment for the initial evolution of miRNAs by favouring their integration into transcriptional networks, while providing stronger expression constraints in the long term. We present data that young intragenic miRNAs have a richer set of target genes, are enriched in neural tissues and are less associated with diseases than intergenic ones. Finally, we discuss possible functional implications associated with miRNA evolution and their genomic location.

## Results

### Intragenic miRNAs are mostly embedded within old genes

**Figure 1:**
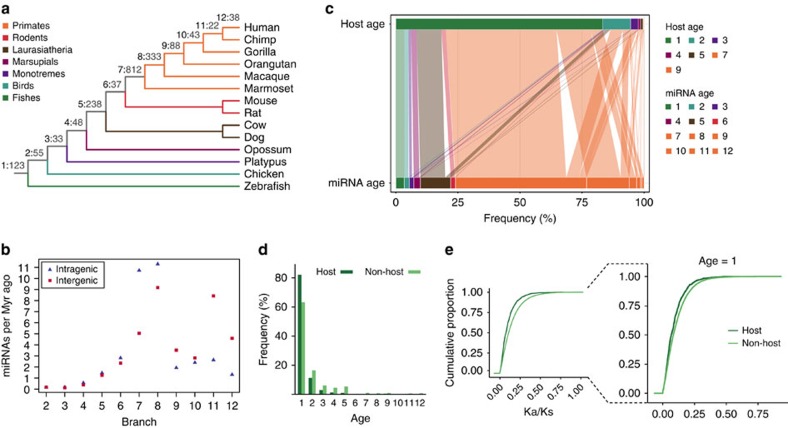
Evolutionary ages of human inter- and intragenic miRNAs and their host genes. (**a**) MiRNA distribution along the vertebrate phylogenetic tree. Numbers in grey indicate the amount of miRNAs (miRBase 20) emerged in each phylogenetic branch (1–12). (**b**) Number of human inter- and intragenic miRNAs across the vertebrate lineage. Numbers of miRNAs per million years (Myr ago) were calculated by the ratio of inter- or intragenic miRNAs emerged in each branch to the time elapsed from the previous branch. For example, the gain rate of intergenic miRNAs in branch 2 (chicken) is given by *N*_inter_/*D*_b12−b1_−*D*_b12−b2_, where *N*_inter_ is the number of intergenic miRNAs emerged in branch 2; *D*_b12−b1_ is the divergence time between branches 12 (human) and 1 (fish) and *D*_b12−b2_ is the divergence time between branches 12 and 2. Divergence times were obtained from timetree.org. (**c**) Age relationships among intragenic miRNAs and their host genes. Horizontal line lengths are proportional to the frequency of miRNAs and host genes of each age. (**d**) Host and non-host genes' frequency according to their ages. Single exon genes were excluded to avoid new gene bias in non-host genes due to excess of retrogenes. (**e**) Ka/Ks cumulative distributions for host and non-host genes. Distribution for old genes (age=1) is shown in detail.

To investigate the influence of host genes and the genomic context on miRNA expression and evolution, we first classified them as inter- or intragenic, depending on the overlapping with coding genes. The set of human miRNAs (miRBase v.20, *N*=1,870) is composed by ∼39% of intergenic and ∼61% of intragenic miRNAs (within 986 coding host genes) of which ∼84% are located on the same strand of their host genes. Intragenic miRNAs predominantly map into introns (90%), whereas those overlapping exons (10%) are mostly (∼80%) found on non-coding regions (5′ and 3′ UTRs). Ages of human miRNAs were inferred using other 13 vertebrate species. According to other studies^25^, 85% of the miRNAs emerged after the split of placental mammals (branches 5–12), whereas we estimate that the bulk of human annotated miRNAs (∼70%) originated in primates (notably in branches 7 and 8) (Fig. 1a). The relationship between the number of homologous miRNAs and gap content of each species genomes shows consistency of miRNA numbers among closest groups, indicating that genome quality did not undermined miRNA identification (Supplementary Fig. 1a). Thereafter, we grouped miRNAs into age classes (1: vertebrates, 2–4: amniotes, 5–6: placental mammals and 7–12: primates), which will be used in subsequent analyses, except if specified elsewhere.

As intragenic miRNAs became highly prevalent in vertebrates, particularly after the split of mammals^9^, we decided to better characterize the dynamics of inter- and intragenic miRNA origination by comparing the amount of each miRNA category along evolutionary branches (Fig. 1a). We observed unequal rates of inter- and intragenic miRNA origination, especially across the primate lineage. The highest peak of intragenic miRNA emergence occurred in branch 7 (*P*<2.2 × 10^−16^, two-sided Binomial test; Fig. 1b), whereas significant excess of intergenic miRNAs appeared in branches 9 (*P*=0.01, two-sided Binomial test), 11 (*P*=0.02, two-sided Binomial test) and 12 (*P*=0.001, two-sided Binomial test) (Fig. 1b). However, variables such as common duplication origin and lack of expression could directly affect these interpretations. Therefore, by merging expressed miRNAs 10 kb apart from each other (see Methods), we indeed observed that intergenic miRNAs seem to be more prone to aggregate into clusters (odds ratio=1.4, *P*=6 × 10^−4^, Fisher's exact test). However, the overall highest rate of intragenic miRNA origination in branch 7 is maintained (*P*<2.2 × 10^−16^, two-sided Binomial test; Supplementary Fig. 1b), and branches 5 and 8 also showed excess of intragenic miRNAs (*P*<0.02, two-sided Binomial test; Supplementary Fig. 1b). Finally, we took advantage of a highly curated data set recently provided by Fromm *et al*.^26^ (http://www.mirgenedb.org, v1.1), which is a re-annotation of miRBase entries based on a set of stringent criteria to exclude non-bona fide miRNAs. Even with less than one third of the original human miRBase entries^26^, the excess of intragenic miRNAs in branch 7 was evident (*P*=0.001, two-sided Binomial test, Supplementary Fig. 1c). Thus, our results reveal that intragenic miRNAs began to prevail at least in placental mammals, whereas most of the human miRNA repertoire was acquired in primates through a substantial accumulation of miRNAs embedded within coding genes.

**Figure 2:**
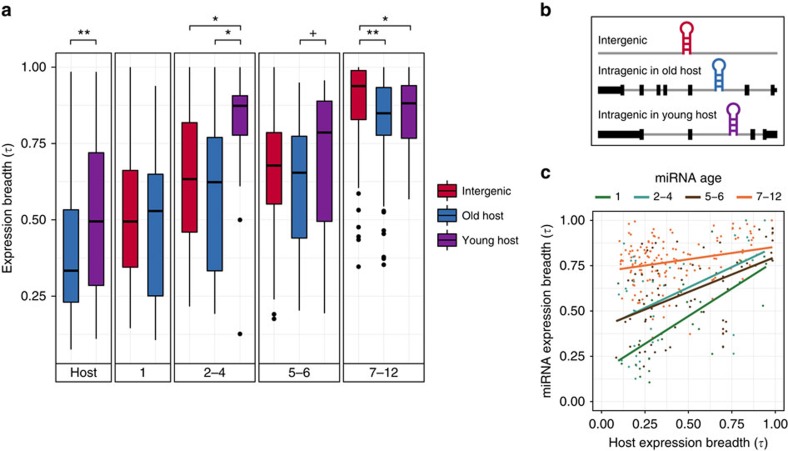
Human miRNA expression breadth according to age and genomic context. (**a**) Box plots of expression breadth calculated using the tissue specificity index (*τ*). Higher *τ* indicates more tissue specificity. First box represents the expression breadth of old (blue) and young (purple) host genes. The subsequent boxes correspond to the expression breadth of intergenic miRNAs (red), intragenic miRNAs within old hosts (blue) and intragenic miRNAs within young hosts (purple), according to miRNAs' age classes (1: vertebrates, 2–4: amniotes, 5–6: placental mammals and 7–12: primates). Significant differences in *τ* were assessed by Mann–Whitney tests (^+^*P*=0.07; *P<0.05; **P<0.001). (**b**) Schematic representation of the miRNA genomic contexts considered in our analyses. (**c**) Correlations between expression breadth of intragenic miRNAs and of their host genes according to miRNA age class. Significant correlations were observed for all ages (age 1: *ρ*=0.67, *P*<0.001; age 2–4: *ρ*=0.36, *P*=0.03; age 5–6: *ρ*=0.47, *P*<0.001; age 7–12: *ρ*=0.20, *P*=0.01, Spearman rank correlations).

To start testing our hypothesis that host gene age impacts the expression and evolution of miRNAs, we accounted miRNA genomic location as: intergenic, intragenic within old host or intragenic within young host (age=1 or age⩾2, respectively, Fig. 1a; according to the studies by Zhang *et al*.^27^,^28^). We observed that a large fraction (∼83%) of host genes is old (Fig. 1c), even after limiting our analysis for clustered and expressed miRNAs (Supplementary Fig. 1d). This suggests that intragenic miRNAs are more likely to arise or become fixed within old genes, Fig. 1d and *P*<0.0001, randomization test or *P*=3.93 × 10^−11^, *χ*^2^-test; Supplementary Fig. 1e). Significant differences were also observed when using gene ages obtained from two alternative dating methods (Supplementary Fig. 2a,b). However, as reported for old genes^22^,^29^, old hosts are longer than young ones (*P*=0.002, Mann–Whitney *U*-test; Supplementary Fig. 1f) due to intron accumulation over time^29^, retroposition origin of young genes^23^ or processing errors and cost in keeping long young genes^30^, for example. In fact, gene length is thought as a conservation predictor as intron burden is related to complexity of gene function and expression^29^,^30^. Moreover the observed maintenance bias in old genes could be a product of insertion bias as those genes have more room to accommodate miRNAs. Yet, even when sampling from all host genes with no significant size difference from young hosts (Supplementary Fig. 1g) we still observed the same proportion of old hosts, similar to the original data set (80%, *P*=0.57, *χ*^2^-test). We adopted an analogous control for expression breadth as old hosts tend to be expressed in more tissues than young ones (Fig. 2 and Supplementary Fig. 1h) and thus could potentially bias miRNA detection within old genes. By randomly sampling from all host genes with no significant expression breadth difference from young hosts (Supplementary Fig. 1i), we still observed the same high frequency of old hosts (81%, *P*=0.76, *χ*^2^-test), suggesting that host gene expression is not the only factor facilitating miRNA fixation in genic regions, but also the ancient origin of host genes. By using the stringent miRNA annotation^26^, the proportion of old hosts remained significantly high (82%, *P*=3.93 × 10^−11^, *χ*^2^-test). Curiously, the oldest age among young host genes was overrepresented (age=2, *P*<0.0001, *χ*^2^-test, Supplementary Fig. 1j). Moreover, similar to old genes^21^, host genes tend to evolve more slowly than non-hosts as shown by lower Ka/Ks values (*P*<2.2 × 10^−16^, Kolmogorov–Smirnov (KS) test; Fig. 1e). This pattern holds even when comparing Ka/Ks distributions only for old genes (age 1) (*P*=3.2 × 10^−6^, KS test, Fig. 1e), indicating that host genes are indeed old and subjected to strong sequence constraints. Therefore, our analyses point to a preferential emergence and fixation of intragenic miRNAs within old genes, irrespective of age estimation method, gene length or expression, leading us to suggest that a selective pressure must underlie this pattern.

### Host gene age and genomic context affect miRNA expression

To investigate the influence of genomic context and host gene age on miRNA expression, we considered our previously defined age classes (1, 2–4, 5–6 and 7–12) and genomic location categories (inter- and intragenic). Then, we determined the expression breadth of miRNAs and host genes using the tissue specificity index (*τ*) (ref. 31) across 12 and 16 tissues or cell types, respectively. Expression level and particularly expression breadth have been well-recognized as correlated with evolutionary rates, meaning that conserved genes are in general highly and broadly expressed^22^,^23^,^32^. Accordingly, we observed that old host genes are more broadly expressed (that is, lower *τ*) than young ones (*P*=1.4 × 10^−6^, Mann–Whitney *U*-test; Fig. 2a). Interestingly, young hosts of the same age are more broadly expressed than young non-host genes (age 2: *P*=0.007, age 4: *P*=0.001, Mann–Whitney *U*-tests; Supplementary Fig. 3a). Also, older miRNAs have higher expression breadth (age 1 versus 2–4, 5–6, 7–12: *P*<0.002; age 2–4 versus 7–12: *P*<2.2 × 10^−16^; age 5–6 versus 7–12: *P*<2.2 × 10^−16^, Mann–Whitney *U*-tests; Fig. 2a). By comparing the expression breadth within same age classes we found that, despite small sample size (*N*=13 for both 2–4 and 5–6), miRNAs emerged in amniotes or placental mammals embedded in young hosts tend to be more narrowly expressed than those within old hosts or located in intergenic regions (age 2–4: young host versus old host—*P*=0.01, young host versus intergenic—*P*=0.01; age 5–6: young host versus old host—*P*=0.07, Mann–Whitney *U*-tests; Fig. 2a, see Fig. 2b for a schematic illustration). In addition, young primate intragenic miRNAs (age 7–12) are more broadly expressed than young intergenic ones, whereas the most pronounced difference lies on miRNAs within old hosts (old host versus intergenic: *P*=1.6 × 10^−7^; young host versus intergenic: *P*=0.004, Mann–Whitney *U*-tests; Fig. 2a). The same trend appears for expression breadth calculation based only on the number of tissues in which miRNAs are expressed (Supplementary Fig. 3b).

The robustness of our observations was verified by recalculating miRNA expression breadth controlling for several variables: (1) removing testis expression data (Supplementary Fig. 3c), as novel genes usually show testis-biased expression^33^ and much of the `promiscuous' transcription is imputed to the permissive chromatin conformation during spermatogenesis^34^. Thus, ruling out the ‘the out of the testis hypothesis', which predicts that new genes primarily expressed in testis would evolve more diverse expression patterns^35^, could be a major factor on the evolution of miRNA expression breadth. (2) Removing brain and cerebellum expression to avoid bias inherent from neural transcriptome complexity^36^ (Supplementary Fig. 3d); (3) clustering miRNAs 10 kb apart (Supplementary Fig. 3e); (4) using miRNA ages obtained from a different study^25^ (Supplementary Fig. 3f); (5) using data generated by the same study (Supplementary Fig. 3g) to reduce potential bias caused by technical and/or biological variability; (6) comparing inter- and intragenic miRNAs of age 7 only (Supplementary Fig. 3h), as intergenic was the most frequent type of miRNAs from ages 9 to 12 (Fig. 1b). (7) testing for bias due to mirtron expression^37^ (Supplementary Fig. 3i), which indicates that host gene ages also affect the expression breadth of intronic miRNAs processed through the canonical pathway. Finally, we evaluated the expression breadth of bona fide miRNAs curated by Fromm *et al*.^26^ and the same patterns were observed: miRNAs within young hosts tend to be more narrowly expressed than others of the same age and young miRNAs within old host genes are indeed more broadly expressed than young intergenic ones (*P*=1.1 × 10^−9^, Mann–Whitney *U*-test; Supplementary Fig. 3j). By using this robust data set, the possibility that expression breadth of old hosts would benefit preferentially non-bona fide miRNAs and that the remaining young ones would simply represent unspecific by-products of host gene expression is rather unlikely. In a consistent manner, none of these above mentioned potential sources of bias changed our main conclusion that miRNA expression breadth is influenced by host gene age. Moreover, this is consistently observed along miRNAs of different ages, implying preferential maintenance over preferential emergence, of intragenic miRNAs within old genes. We therefore interpret such pattern as likely driven by natural selection.

Supporting the notion that miRNA expression breadth is influenced by host gene age, we observed significant positive correlations between expression breadth of miRNAs and their host genes (age 1: *ρ*=0.67, *P*=2.0 × 10^−4^; age 2–4: *ρ*=0.36, *P*=0.03; age 5–6: *ρ*=0.47, *P*=2.0 × 10^−5^; age 7–12: *ρ*=0.20, *P*=0.01, Spearman rank correlations; Fig. 2c). Noteworthy, correlation of young miRNAs is basically explained by old hosts' expression breadth (Supplementary Fig. 4). Furthermore, higher expression level was observed for young intragenic miRNAs within old hosts compared with young intergenic ones when using the MirGeneDB^26^ annotation (*P=*2.34 × 10^−5^, Mann–Whitney *U*-test; Supplementary Fig. 5a–d).

As miRNAs and host genes are often co-transcribed^14^,^18^,^38^, expression breadth correlations between miRNAs and their host genes are expected to be related to co-expression (expression in the same tissues). Then we tested and confirmed that miRNAs and their host genes are more co-expressed than what would be expected by chance (all age classes, except 2–4: *P*<1.0 × 10^−4^, randomization tests; Supplementary Fig. 6), indicating the great contribution of co-expression to the influence exerted by host genes on miRNA expression breadth.

**Figure 3:**
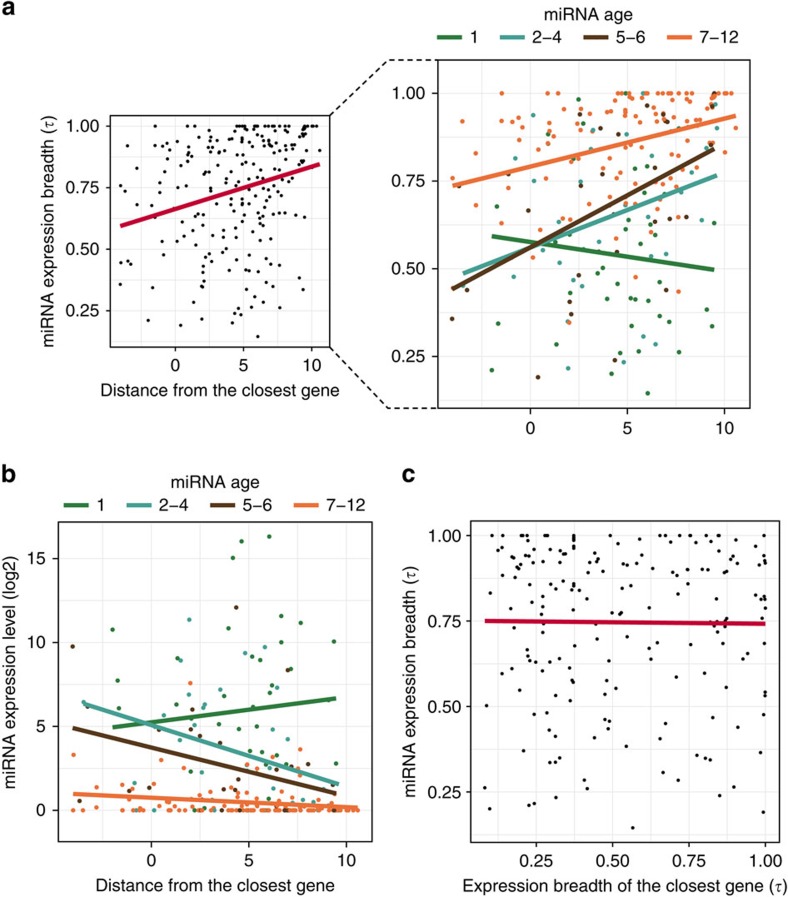
Intergenic miRNA expression with respect to their closest neighbouring genes. (**a**) Correlation between miRNAs' expression breadth and distance to closest genes (*ρ*=0.31, *P*<0.001, Spearman rank correlation), showing that intergenic miRNAs tend to be more broadly expressed when closer to coding genes. Correlations by each miRNA age class are shown in detail (all except age 1: 0.35<*ρ*<0.5; *P*<0.05). (**b**) Correlations between miRNA expression level and distance to closest genes by each miRNA age class. The expression level is given by the median of miRNA expression level in the tissues in which they are expressed. Significant correlations were observed for all ages (except for age 1) (−0.36<*ρ*<−0.25; *P*<0.05). (**c**) Correlation between expression breadth of miRNAs and their closest neighbours in the same strand. No significant correlation was observed (*ρ*=−0.03, *P*=0.6). For these analyses, merged miRNAs apart up to 10 kb from each other were used.

We next asked whether the expression of intergenic miRNAs is also affected by their genomic context by focusing on the neighbouring coding genes. Intergenic miRNAs are distant from dozens of bases to >1.5 Mb (median=34 kb; Supplementary Fig. 7a), whereas ∼33% are up to 10 kb apart from their closest neighbours. Cabili *et al*.^39^ found a similar pattern for lincRNAs with respect to their protein-coding neighbours. Interestingly, expression breadth tends to be higher as intergenic miRNAs get closer to coding genes (*ρ*=0.31, *P*=1.84 × 10^−6^; Spearman rank correlation; Fig. 3a) as significant positive correlations to gene proximity were found for miRNAs emerged after the fish–bird split (age 1: *ρ*=−0.13, *P*=0.30; age 2–4: *ρ*=0.36, *P*=0.03; age 5–6: *ρ*=0.48, *P*=0.006; age 7–12: *ρ*=0.36, *P*=6.37 × 10^−5^; Spearman rank correlations, Fig. 3a). Expression level also tends to be higher with gene proximity (age 1: *ρ*=0.16, *P*=0.30; age 2–4: *ρ*=−0.36, *P*=0.03; age 5–6: *ρ*=−0.36, *P*=0.04; age 7–12: *ρ*=−0.25, *P*=0.004; Spearman rank correlations, Fig. 3b). In contrast to results found for intragenic miRNAs and their host genes, expression breadth of intergenic miRNAs and of their closest neighbours is not correlated (Fig. 3c), even when considering only miRNAs within 10 kb from their neighbours or choosing closest genes downstream and in the same strand orientation as the miRNAs (Supplementary Fig. 7b–d). In addition, we found no biases related to the ages of neighboring genes nor to the ages of their closest intergenic miRNAs. Therefore, gene proximity seems to affect the expression of intergenic miRNAs (with age⩾2), but not coordinately like host genes do on intragenic miRNAs. An alternative explanation for those correlations might be related to the transcriptional activity favoured by the open chromatin in gene neighbourhood^39^,^40^, instead of co-regulation.

In summary, here we show that host gene age affects the expression breadth of intragenic miRNAs. Therefore, young intragenic miRNAs, which were expressed in more tissues than young intergenic ones, would suffer strong influence by old host genes. Expression level seems to be not affected in the same degree. On the other hand, both expression level and breadth of intergenic miRNAs are subjected to gene proximity, possibly through an effect of the surrounding chromatin state.

### Differential expression constraints on intragenic miRNAs

**Figure 4:**
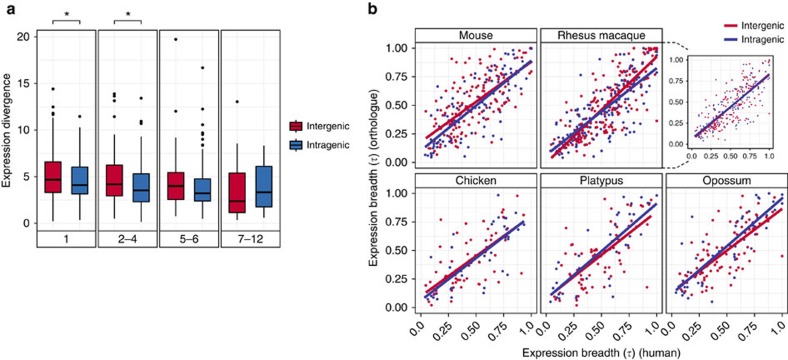
Interspecies analyses of miRNA expression. (**a**) Expression divergence between human miRNAs and their orthologues in five species (chicken, platypus, opossum, mouse and rhesus macaque). The expression divergence was calculated by means of Euclidean distances of expression levels across five tissues (brain, cerebellum, heart, kidney and testis) for each pair of orthologues. Box plots represent the distribution of pooled distances for each pair according to miRNA age class. Significant differences were assessed by Mann–Whitney *U*-tests (*P<0.02). (**b**) Spearman's correlations between expression breadth of human miRNAs and their orthologues with respect to their genomic context (inter- and intragenic). Correlations are as follows: Chicken: intergenic: *ρ*=0.70, intragenic: *ρ*=0.78; Platypus: intergenic: *ρ*=0.68, intragenic: *ρ*=0.83; Opossum: intergenic: *ρ*=0.76, intragenic: *ρ*=0.85; Mouse: intergenic: *ρ*=0.69, intragenic: *ρ*=0.81; Rhesus macaque: intergenic: *ρ*=0.86, intragenic: *ρ*=0.79; Rhesus macaque excluding miRNAs of age 7 (shown in detail): intergenic: *ρ*=0.79, intragenic: *ρ*=0.81 (all *P*<1.0 × 10^−9^). To assess the significance of the difference between two correlation coefficients, we used the Fisher *z* transformation. Significant differences between inter- and intragenic correlations were observed for Platypus (one-tailed *P*=0.02), Opossum (one-tailed *P=*0.05) and Mouse (one-tailed *P*=0.01). A minimum of 1 c.p.m. (counts per million) in at least one tissue was adopted as expression threshold for both analyses (**a**,**b**).

Gene expression differences across species are thought as one of the major determinants of phenotypic diversity^41^. Although the real contribution of natural selection in shaping expression levels is debatable^42^, it is clear that gene regulation evolves under stabilizing selection for large gene sets, implying in lower expression variation within and between species^43^. In this sense, we asked whether expression differences between species behave similarly for inter- and intragenic miRNAs. To do so, we assessed miRNA expression levels from five tissues (brain, cerebellum, heart, kidney and testis) for human, rhesus macaque, mouse, opossum, platypus and chicken. Then, we determined the expression divergence between human miRNAs and their orthologues by means of Euclidean distances. Notably, we found that older intragenic miRNAs showed slight significant lower expression divergence than intergenic ones (age 1: *P*=0.019, age 2–4: *P*=0.017, Mann–Whitney *U*-tests, Fig. 4a). Moreover, expression breadth comparisons between human and orthologous miRNAs revealed greater correlations for older (that is, excluding those of age 7 in rhesus macaque) intragenic miRNAs relative to intergenic ones (significant differences were assessed by Fisher *z* transformations and were observed for platypus (one-tailed *P*=0.02), opossum (one-tailed *P=*0.05) and mouse (one-tailed *P*=0.01), Fig. 4b).

We next evaluated whether expression constraint for older intragenic miRNAs is accompanied with differential conservation at sequence level. Distributions of PhyloP scores^44^ across miRNA precursors finely agree with our age class definitions, as the older the miRNA age class is the higher the phyloP scores are (Supplementary Fig. 8a). Overall, no significant differences of sequence conservation were evident, except for higher scores of young intragenic miRNAs (age 7–12) compared with intergenic ones (Supplementary Fig. 8a). Nevertheless, such difference is likely related to surrounding genomic regions, indicated by higher phyloP scores for intragenic random background (Supplementary Fig. 8a). In regard to the most preserved sequence—the seed region^45^—no differences between inter- and intragenic miRNAs were found, although greater sequence conservation with respect to whole precursors was evident even for young miRNAs (Supplementary Fig. 8b). These results suggest that, in the long term, intragenic miRNAs might be subjected to stronger expression constraints than intergenic ones, but apparently not related to sequence constraint itself. One possible scenario is the consequence of a tighter regulatory control influenced by the genomic environment of old host genes (see Discussion).

### Functional connections between miRNAs and genomic location

**Figure 5:**
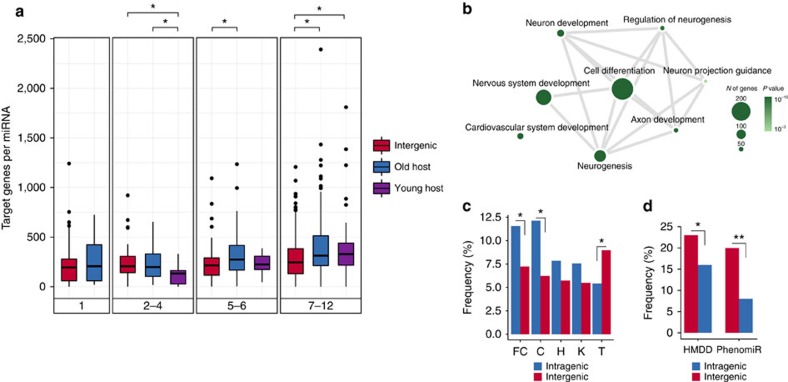
Functional analyses of inter- and intragenic miRNAs. (**a**) miRNA target analysis. Box plots for the distributions of the number of target genes per miRNA predicted using TargetScan 7.0 algorithm. Significant differences were assessed by Mann–Whitney tests (*P<0.05). (**b**) Enriched biological processes from Gene Ontology (GO) for host genes. Associated terms are connected by grey lines; size and colour of the circles are proportional to the number of genes in each category and to the corrected *P* value, respectively. (**c**) Enrichment of young inter- and intragenic miRNAs in frontal cortex (FC), cerebellum (C), heart (H), kidney (K) and testis (T). The *y*-axis represents the frequency of young miRNAs expressed in each tissue relative to the total of young miRNAs of each category (inter- or intragenic). Overrepresentation of intragenic miRNAs relative to intergenic and *vice versa* was assessed by Fisher's exact test (*P<*0.*05, ^+^*P=*0.06). For this analysis, we considered the data set where miRNAs distant up to 10 kb from each other were merged into clusters. (**d**) Young miRNAs in disease-associated data sets provided by PhenomiR and HMDD. Frequencies represent the number of young miRNAs (age 7–12) associated with diseases relative to the total of young miRNAs of each category (inter- and intragenic). (*P<0.05, **P<0.001, Fisher's exact test).

Differences between inter- and intragenic miRNAs motivated us to explore possible functional aspects related to these two categories. For example, we have shown that young intragenic miRNAs are more broadly expressed than young intergenic ones (Fig. 2a). We then supposed that such intragenic miRNAs would target more genes, as they are apparently exposed to more diverse cellular contexts. Target prediction indicates that young intragenic miRNAs have a richer target set compared with young intergenic ones (age 7–12: intragenic in old host versus intergenic—*P*=0.003, intragenic in young host versus intergenic—*P*=0.01, Mann–Whitney *U*-tests; Fig. 5a). Even when considering the highly curated miRNA annotation^26^ the same pattern was observed (Supplementary Fig. 9). Although it is consensus that target prediction may lead to unreliable predictions, we speculate that young miRNAs emerging within coding genes, especially those within old hosts, due to the higher expression breadth, may regulate a wider target repertoire, though more powerful computational and experimental approaches are certainly required to corroborate this idea.

Clues about functional roles of intragenic miRNAs might come from host gene functions. Thus, we carried out a functional enrichment analysis with the set of host genes. Interestingly, host genes are particularly associated with neuronal functions (Fig. 5b, Supplementary Data 1 and 2). We observed that genes of neural functions tend to be longer than other genes, then we tested for gene size as a potential source of bias, however, no enrichment of neural functions was observed for randomly sampled genes with similar sizes of neural genes (Supplementary Fig. 10). Tissue expression enrichment analysis using DAVID also revealed a marked overrepresentation of host genes in brain (Benjamini corrected *P*=1.1 × 10^−11^). Next, we tested whether young inter- and intragenic miRNAs are unevenly represented in a particular tissue. We observed that the set of young intragenic miRNAs is overrepresented (relative to intergenic) in neural tissues (brain: *P*=0.01, cerebellum: *P*=0.002, Fisher's exact tests; Fig. 5c), which is in agreement with previous reports showing brain-specific expression of young miRNA families^9^ and indicates functional relationships with their host genes. Thereby, the connection of neuronal roles played by host genes with the overrepresentation of primate-specific intragenic miRNAs in brain and cerebellum suggest a joint contribution to the evolution of regulatory networks in neural tissues. On the other hand, the set of young intergenic miRNAs (relative to intragenic) is slightly overrepresented in testis (*P*=0.06, Fisher's exact test; Fig. 5c). This is perhaps linked to the dual explanation for testis-biased expression of young genes, which advocates for leaky expression facilitated by chromatin remodelling during spermatogenesis^34^ or action of selective pressures due to sexual conflict^9^,^33^.

Considering that miRNA expression variation might be linked to their hosts' regulatory activity^18^, and many diseases are associated with abnormal miRNA expression, we envisioned a distinct interplay of inter- and intragenic miRNAs with human diseases. To verify this possibility, we used information provided by HMDD^46^, a curated database of disease-associated miRNAs. Curiously, a greater proportion of young intergenic miRNAs (relative to intragenic ones) was associated with diseases (age 7–12: *P*=0.02, Fisher's exact test; Fig. 5d). Similar results were obtained using PhenomiR^47^, another database that compiles information about miRNAs with altered expression in diseases (age 7–12: *P*=1.5 × 10^−6^, Fisher's exact test; Fig. 5d). MiRNAs of ages 1, 2–4 and 5–6 grouped together also present an overrepresentation of intergenic miRNAs (*P*<0.03, Fisher's exact test). An explanation for this might be related to how miRNAs respond to perturbations on their transcriptional activity. As regulatory regions of host genes and intragenic miRNAs are commonly shared^38^, it is possible that intragenic miRNAs, especially the younger ones, are ‘safeguarded' by the tight transcriptional control of old host genes, resulting in less variation in miRNA expression (see Discussion).

## Discussion

Emergence of miRNAs overlapping transcriptional units has been largely documented^9^,^14^,^15^,^16^,^17^, however, many of the functional and evolutionary consequences of this genomic organization are still unknown. In this study, we depicted evolutionary patterns of human miRNAs in light of the genomic context and host gene influence. By examining miRBase annotated miRNAs, we observed that intragenic miRNAs started to outnumber intergenic ones in placental mammals. Coincidently with the burst of miRNA origination in primates, the peak of intragenic miRNA acquisition occurred after the rodent–primate split, specifically in branches 7 and 8, whereas taking into account the recent proposed miRNA annotation^26^ we observed the excess of intragenic miRNAs in branch 7 (primates).

Our analyses revealed that intragenic miRNAs are likely to emerge within old genes, pointing to important functional and evolutionary implications. We found that old host genes are indeed more broadly expressed than young ones^22^,^23^,^32^, having stronger signal of sequence constraint and are probably subjected to strong purifying selection (lower Ka/Ks ratios), even when compared with old genes not harbouring miRNAs. Here we showed that host gene age directly affects the expression breadth of embedded miRNAs. Specifically, we observed that miRNAs within young hosts tend to be more narrowly expressed than miRNAs of the same age within old hosts or intergenic ones. We also found that primate miRNAs embedded in old host genes are more broadly expressed than their intergenic counterparts. We emphasize that the pattern in which old gene environment leads to increase of expression breadth and levels of hosted miRNAs is consistently observed along different evolutionary ages, not only for young miRNAs. Therefore, it is unlikely that such mechanism exclusively underlies the short period of ‘proto-miRNA' emergence. One could argue that the excess of young intragenic miRNAs in old host genes would be solely a consequence of higher chances to be expressed and processed by miRNA biogenesis machinery, as old genes are more highly and broadly expressed^22^,^23^,^32^. However, our data showed that: (i) host expression does not account entirely for the excess of old host genes; (ii) by excluding several putative misannotated miRNAs (or ‘proto-miRNAs') we still obtained the same high proportion of old host genes and significant expression breadth differences among young inter- and intragenic miRNAs and (iii) even older miRNAs, as those emerged in branches 2–4 and 5–6, that in principle should have acquired their promoters more frequently^38^, show expression breadth differences if located in old or young hosts. Together this indicates that host environment also impacts miRNA expression in the long run. Therefore, we suggest that such patterns should be explained by invoking natural selection, rather than solely a by-product effect of host's expression.

In summary, the advantage provided by old hosts to miRNAs has evolutionary consequences in terms of miRNAs fixation in the long run and suggests the generality of its effects. Such age consistent scenario is contrary to the hypothesis that the observed pattern is a consequence of a data set enriched with ‘proto' or non-functional miRNAs. Nevertheless, we observed robust host effect even for a recent highly stringent miRNA annotation^26^.

Therefore, we propose that ‘proto-miRNAs' may originate without large biases regarding genomic location, but the genomic context (especially host gene age) influences the chances of fixation. In this sense, young miRNAs, like young coding genes, show inherent low expression level, which can later on reach higher expression levels and become functional after retained by the sieve of natural selection^48^. Despite their initial low expression levels, such new genes are not necessarily functionally negligible. New genes can indeed be quickly integrated into transcriptional networks and even become essential^49^, while this process might be even easier for miRNAs^12^. Hence, it does not mean that all these young miRNAs are truly functional, but they represent a rich ground in which natural selection can act on and drive functionality in the future^12^.

Our study shows that miRNA tissue-expression range is correlated with the expression breadth of host genes, which is tied to co-expression of miRNA–host pairs. Although miRNA–host expression discrepancies are usually due to the use of independent intronic promoters^38^,^50^ or differential miRNA stability^51^ our results emphasize a co-regulation scenario, particularly for recent miRNAs, which have been recently suggested to be more likely regulated by shared promoters with their host genes^38^. In addition, the chromatin state encompassing the genomic region of host genes may also exert influence on the time and place of intronic miRNA expression, including those derived from spliced introns (processed through the canonical or mirtron pathways) or those independently transcribed from their own promoters. Analogously, we showed that intergenic miRNA expression (except for oldest ones) seems to be affected by neighbouring genes, revealed by increase of both expression breadth and levels in gene proximity. In contrast to intragenic miRNAs and their host genes, we found no evidence of co-regulation between intergenic miRNAs and neighbouring genes, leading us to speculate that open chromatin facilitates transcription of proximal miRNAs, as similarly outlined for lincRNAs close to coding genes analysed by Cabili *et al*.^39^.

Given that most of the human miRNA repertoire resides within old genes, how does such an ancient genomic context impact the expression and evolution of intragenic miRNAs? Recently, Popadin *et al*.^52^ analysed essential gene properties associated with gene age, claiming ‘gene age can be an evolutionary proxy for the level of functional constraints of a gene'. They showed that cis-eQTLs of old genes have lower effect size, are less significant, farther from the transcription start site and affect fewer tissues than cis-eQTLs of young genes, implying in an increase of expression constraints associated with old genes. Consistent with this evolutionary framework, we found evidence that old and middle-aged intragenic miRNAs, compared with intergenic counterparts, show lower expression divergence and greater expression breadth correlations between species. Moreover, we observed that younger intragenic miRNAs are underrepresented in miRNA disease-associated data sets. As those associations are largely inferred from abnormal miRNA expression, we would not expect that most of obtained miRNA disease associations are explained by seed sequence mutations or editing. Therefore, assuming that many miRNAs are under their hosts' regulatory control, we could speculate that cis-eQTLs of host genes may affect the expression of embedded miRNAs. Thereby, a presumable tight regulatory control driven by old host genes would confer stronger expression constraints to co-regulated older intragenic miRNAs, while limiting the expression variation of younger ones in an unstable environment, such as in diseases. A deeper investigation mapping eQTLs of miRNAs and host genes might bring novel insights on that.

One finding of our work is that young intragenic miRNAs seem to capture the higher expression breadth and levels from their old hosts. According to the model proposed by Lyu *et al*.^13^, the maintenance of newly emerged miRNAs depends on their integration into transcriptional networks. A possible benefit caught by young miRNAs residing in old genes would be an effective incorporation into regulatory programmes impelled by the spatiotemporal control of their hosts' expression. Since these miRNAs are expressed in a wider range of tissues, they can reach more targets, as our target prediction analysis suggested, possibly speeding up the settlement of miRNA–target relationships that can be shaped by natural selection over time. One may argue that a vast target repertoire regulated by newly emerged miRNAs could lead to deleterious effects. However, at an early evolutionary phase it is enticing to assume that such miRNAs rarely are expressed enough to cause strong changes on fitness^7^,^53^. Even for some conserved and highly expressed miRNAs, perturbations on their expression levels produce subtle consequences^54^. In addition, experimental analysis showed constraints even for non-conserved target sites, suggesting that they are likely functional at least during an initial evolutionary period^13^,^55^. In this sense, our argument fits well with the role of miRNAs in canalization^53^,^56^,^57^. Under this principle, new miRNAs would mainly act as expression buffers thus reducing expression noise. Young miRNAs would ultimately serve as stabilizers of genetic networks, where weak constraints on specific miRNA–target interactions are expected to occur at least initially^53^. Here we highlight that the broader expression of novel miRNAs emerged from ancient intronic loci would contribute to this process, besides the potential functional innovation introduced by specific miRNA–target regulation.

Several primate-specific miRNAs were detected in human and chimpanzee brains and their roles in establishing part of the cellular diversity in this tissue have been suggested^58^. We add that young intragenic miRNAs and their host genes would be involved in this process, as we observed functional connections such as the overrepresentation of primate intragenic miRNAs in brain and cerebellum parallel to enrichment of host genes expressed in brain and their involvement with neuronal functions. Although neural function enrichment might be influenced by number of studies and annotation bias, such association makes biological sense because of our Gene Ontology (GO) independent observation that young intragenic miRNAs are enriched in neural tissues and previous works showed neural expression of young miRNAs^9^,^58^. In agreement, it is believed that neuronal miRNAs target more coding genes than non-neuronal miRNAs, and target genes of human-specific miRNAs are more associated with neuronal functions^59^. A remarkable example is the human-specific miR-941 hosted by *DNAJC5*. This host gene is repressed by miR-941, as well as two direct interacting partners of the protein encoded by *DNAJC5* (ref. 60). Based on *DNAJC5* and its partner's functions, miR-941 was suggested to participate in neurotransmitter signalling^60^. Recapitulating the key properties of old genes, they are enriched in complex regulatory networks and have higher connectivity^52^. Thus, we might think that miRNAs emerging in old neural hosts would buffer such complex networks in neural tissues by regulating common target sets in synergistic or antagonistic ways^15^,^20^,^61^. Of note, we inspected putative novel miRNAs annotated by Londin *et al*.^62^ and observed similar patterns; the excess of intragenic miRNAs, sense strand orientation bias and host gene expression overrepresented in brain. Conversely, young intergenic miRNAs might have contributed to buffering testis-associated regulatory programmes, suggested by their overrepresentation and higher expression levels in testis. Indeed, rapidly evolving clusters of primate-specific miRNAs linked to X chromosome, which we noticed that are intergenic, were found predominantly expressed in testis^9^,^63^,^64^, targeting genes related to sperm maturation and epididymal morphology^64^.

In conclusion, we provide compelling evidence that host gene constraints and genomic context exert strong influence on miRNA expression and evolution. Most importantly, we pose that host gene age is a key property in shaping the expression patterns of intragenic miRNAs, relating to miRNA expression constraints in the long run, while promoting higher expression breadth for young miRNAs. Once miRNA expression is affected, its evolutionary fate in terms of target interactions would also be impacted. We propose that intronic exaptation^16^ from the ancient and transcriptionally favourable environment of old host genes could boost the functionalization of young miRNAs as canalizing agents, at least during their initial adaptive phase^13^. Noteworthy, recently emerged miRNAs of vertebrate species other than humans are also mostly intragenic compared with older ones^9^. Thus, depending on the genomic context in which miRNAs arise, it can offer a suitable environment for adaptive selection on new miRNAs.

## Methods

### Annotation of human intragenic miRNAs and host genes

Human miRNA precursors downloaded from miRBase (release 20) were classified as intragenic if their genomic coordinates overlapped to protein-coding genes retrieved from Ensembl (release 71) and as intergenic otherwise. Intragenic miRNAs were further classified in sense or antisense orientation with respect to overlapping genes (host genes) and then categorized as intronic or exonic, depending on the overlapped gene region. The longest transcript of the host gene was used as the reference. For subsequent analyses, we used a strict definition of intragenic miRNAs, including solely those in sense orientation to host genes. Intersections between genomic features were performed using the BEDTools suite v2.17.0. For specific analyses, we merged miRNAs apart up to 10 kb from each other. The cluster was considered as a single unit represented by a randomly selected miRNA. We chose 10 kb as previous studies have indicated this as a reasonable cutoff for grouping miRNAs into clusters^65^.

### miRNA age assignment

To assess miRNA ages across the vertebrate lineage, we searched for orthologues of the human miRNA precursors in other 13 genomes obtained from UCSC genome browser, representing primates (chimpanzee: panTro4; gorilla: gorGor3; orangutan: ponAbe2; rhesus macaque: rheMac3; marmoset: calJac3), rodents (mouse: mm10; rat: rn5), Laurasiatheria (cow: bosTau7; dog: canFam3), marsupials (opossum: monDom5), monotremes (platypus: ornAna1), birds (chicken: galGal4) and fishes (zebrafish: danRer7). We used the strategy employed by Hu *et al*.^60^,^66^, with few modifications. First, we retrieved the reciprocal best hits using BLAT (parameters: stepSize=5, -repMatch=2253 -minScore=0 -minIdentity=0), BLASTN (parameters: -word_size 8, -evalue 1e-05) and LiftOver (default parameters), requiring a minimum of 70% and a maximum of 130% of the query sequence length. Next, an orthologue was assigned if the retrieved genomic region was supported by at least two methods. The miRNA mature sequences were identified by aligning the orthologous sequences with the human miRNA precursors using CLUSTALW (default parameters). To take advantage of the existing miRNA information, for orthologues that had overlapping miRBase entries in the same transcriptional orientation and in at least 50% of the region length, we used the official precursor and mature annotations. Finally, the age of a particular miRNA was designated by numbers in ascending order along the species tree, reflecting the most ancient group in which an orthologue was found.

### Gene age assignment

Protein-coding gene ages were kindly provided by Zhang, which adjusted the dating method employed in the studies by Zhang *et al*.^27^,^28^ for a more recent version of the human gene annotation (Ensembl v.71). Briefly, the method relies on finding a human locus with a best reciprocal syntenic alignment in UCSC genome-alignment files, taking into account the conservation of neighbouring genes. A more detailed explanation can be found in the original reports. Gene ages were then reassigned to our species tree, allowing us to parallel the ages of miRNAs and protein-coding genes. The species used to define gene ages are listed in Fig. 1a. To confirm some of our results, we also used two other alternative data sets to infer gene ages, which are detailed in Supplementary Figs 2a,b and 11 (species list obtained from Ensembl).

### Statistical analysis of host gene ages

To verify whether the emergence of intragenic miRNAs is biased towards the age of host genes, we compared the observed proportion of old hosts (age=1, see Fig. 1a) with the expected proportion obtained from a null distribution generated by random sampling 10,000 equal-sized sets of genes just as or older than the sets of miRNAs owing a particular age. We assumed that a host gene appeared before the miRNA, thus we adopted this procedure rather than simply compare the proportion of old hosts with the whole set of human genes. We removed 13 doubtful cases where the host gene was assigned as younger than the miRNA. The empirical *P* value was calculated as the proportion of old genes greater or equal to the observed proportion of old hosts. Alternatively, the average of the null distribution was taken as the expected proportion of old genes and the statistical difference to the observed proportion of old hosts was assessed using the *χ*^2^-test.

### Ka/Ks data

We collected the Ka and Ks values from Ensembl using the human-mouse orthologues. Values greater than 1 were discarded.

### miRNA expression

We collected human small RNA-seq data sets from seven studies publicly available at NCBI Gene Expression Omnibus (GEO) under the accession numbers: GSE46622, GSE33858, GSE47720, GSE37686, GSE32493, GSE31617 and GSE19812. We also used data from the study of Meunier *et al*.^9^ (accession id: GSE40499), which contains data from 5 tissues (frontal cortex/brain, cerebellum, heart, kidney and testis) for 6 species (human, rhesus macaque, mouse, opossum, platypus and chicken), totalizing 12 different tissues or cell types for humans. We only used data sets providing the fastq files and from normal samples. After adaptor removal with FASTX-Toolkit, reads >16 nt were mapped to the respective genomes with Bowtie version 1.0.0, requiring perfect matches (-v 0 -a --best --strata) in no more than 10 different loci (-m 10). MiRNA expression was computed by the sum of the reads entirely overlapping genomic coordinates of the mature miRNAs. To account for alternative precursor processing, we allowed three extra nucleotides at the 3′ end of the mature sequence, while the 5′ end was retained to preserve the seed region. As some identical mature miRNAs can be derived from distinct precursors, multiple mapping reads were divided by the corresponding number of loci. Read counts for each mature miRNA were normalized across samples using the EdgeR package 3.4.2. In some analyses, we used the data set from Meunier *et al*.^9^ separately, so the normalization step was also performed without using the input from the other samples. For downstream analyses, we considered the precursor expression as given by the most highly expressed (regarding the median across all samples) mature miRNA (5p or 3p), setting a threshold of 1 c.p.m. in at least one tissue or cell type. In addition, to account for miRNA families, where identical mature sequences can be produced from different precursors, the mature expression was considered just once by choosing the oldest precursor. Thus, our data set is composed by miRNAs expressed at reliable levels, represented by a single entity and weighted for potential biases from miRNA families.

### Gene expression

The expression levels of human protein-coding genes were obtained for 16 tissues available in the Illumina Human Body Map 2.0 data set, downloaded from EBI ArrayExpress (acession: E-MTAB-513), and for 5 human tissues (brain, cerebellum, heart, kidney and testis) from the data set of Brawand *et al*.^67^, downloaded from GEO (acession: GSE30352). RNA-seq reads were mapped to the human genome (hg19) with TopHat v2.0.8 with default parameters using gene annotations provided by Gencode v16. Alignments were filtered with SAMtools, requiring a minimum mapping quality of 20 (-q 20). Normalized gene expression (FPKM (fragments per kilobase per million of mapped reads)) was calculated by Cufflinks v2.2.1. Only genes with FPKM>1 in at least one tissue were considered for further analyses.

### Expression breadth

To determine the expression breadth of human miRNAs and protein-coding genes, we used the tissue specificity index (*τ*) developed by Yanai *et al*.^31^, adopting a log transformation of the normalized expression values (adding 1 to deal with values<1). The tissue specificity index ranges from 0 to 1, where values closer to 0 indicate broad expression and values closer to 1 indicate narrow expression (that is, more tissue-specific expression). For comparison purposes, narrow expression (*τ*⩾0.7) in our data indicates that miRNAs and coding genes are expressed in a median of 5 (out of 12) and 3 (out of 16) tissues, respectively. Broad expression (*τ*≤0.3) corresponds to miRNAs and genes expressed in 12 and 16 tissues, respectively. Significant differences were assessed by Mann–Whitney *U*-tests. Correlations between expression breadth of intragenic miRNAs and their host genes, and of intergenic miRNAs and their closest genes were calculated using Spearman's rank correlation tests.

To examine whether the correlations between the expression breadth of intragenic miRNAs and host genes were related to co-expression, expression data of miRNAs and host genes available for the same tissues (brain, cerebellum, heart, kidney and testis) from the data sets of Meunier *et al*.^9^ and Brawand *et al*.^67^ were used to perform a randomization test. For a given miRNA–host pair, we sorted tissues by expression level and computed the proportion of tissues wherein a miRNA is expressed in the same rank order of its host gene. The average of proportions was compared with a null distribution generated by shuffling the tissue rank order 3,000 times. We adopted this procedure, rather than a correlation method, due to the intrinsic discrepancies of the expression levels and breadth among older and younger miRNAs, and also due to the small sample size (five tissues). As older miRNAs are more highly and broadly expressed, their expression tend to better correlate with the expression of the host genes (which also tend to be older). Using a randomization test, we weight these disproportions by ranking the order of the tissues in which the miRNA is expressed.

### miRNA expression divergence

Expression divergence between human miRNAs and their corresponding orthologues in five species (rhesus macaque, mouse, opossum, platypus and chicken) were calculated by using Euclidean distances^68^. The normalized expression values (log2 transformed) across a matrix of five tissues (brain, cerebellum, heart, kidney and testis) were used to determine the distances, requiring a minimum expression of 1 c.p.m. in at least one tissue for each orthologue pair.

### Conservation analysis of the miRNA sequences

We analysed the sequence conservation of human miRNA precursors using the base-wise phyloP scores^44^ downloaded from the UCSC genome browser. PhyloP computes conservation or acceleration (faster evolution) under an expected neutral model of evolution in a base-wise manner. Positive scores indicate conservation and negative scores indicate fast-evolving sites. Scores compiled from the 100-way vertebrate alignments were used for miRNAs conserved beyond mammals (age classes 1 and 2–4, see Fig. 1a), the 46-way placental mammals for those originated in placental mammals (age class 5–6) and the 46-way primate alignments for primate-specific miRNAs (age class 7–12). The phyloP score of each precursor was determined by the average of the individual base scores. To estimate the random background of a particular intragenic miRNAs, we obtained the average scores for 100 randomly selected intronic regions (with equal sizes of the miRNAs) belonging to its host gene. For intergenic miRNAs, we computed the average scores of 100 random intergenic regions within a window of 10 kb up or downstream of the miRNA. We also computed the phyloP scores of the flanking regions adjacent to the miRNAs for comparison and similar results were obtained. For the seed score assessment, we used our set of expressed miRNAs, which is controlled for redundant mature sequences and it is represented by the most expressed mature miRNA.

### miRNA target prediction

To predict targets of human miRNAs, we ran the TargetScan 7.0 algorithm using the 3′ UTR sequences of protein-coding genes (considering the longest isoform per gene) provided by the TargetScan website (http://www.targetscan.org). This latest version of the algorithm is claimed to predict targets with comparable efficiency to high-throughput experimental approaches^69^. To get a more reliable set of targets, we restricted the predictions to genes with 7mer-m8 or 8mer site types and with individual context+ score<−0.25. To make the number of target genes more comparable among miRNAs containing one and/or two annotated mature sequences and different number of family members (defined by sharing the same mature), we restricted the analysis to our data set of expressed miRNAs, which is controlled for these issues. As the current set of human annotated miRNAs is largely composed by young entries, we did not rely on evolutionary target site conservation. Differences in the number of target genes per miRNA were assessed using Mann–Whitney *U*-tests.

### miRNAs associated with diseases

To test for the over or underrepresentation of inter- and intragenic miRNAs associated with diseases, we performed Fisher's exact tests using the data sets provided by the HMDD v2.0 (ref. 46) and PhenomiR 2.0 (ref. 47) databases. To be more accurate with the period in which these data sets were released, we used different versions of miRBase (19 and 17, respectively) to obtain the number of inter- and intragenic miRNAs.

### Functional enrichment analysis of the host genes

We performed a GO analysis of the biological processes enriched in the set of host genes using AmiGO 2 (http://amigo.geneontology.org/amigo) and using the Bonferroni correction at *P*<0.05. The list of enriched terms associated with host genes are provided in Supplementary Data 1. Then, we used REViGO (http://revigo.irb.hr/) to summarize redundant GO terms. The enrichment analysis provided by DAVID 6.7 (https://david.ncifcrf.gov/) led to very similar results (Supplementary Data 2). We also used DAVID to evaluate the enrichment of host genes in tissue expression. Significant *P* values provided by DAVID's Ease score were considered at *P*<0.05 by adopting the Benjamini–Hochberg multiple testing correction.

### Data availability

Computer codes and the data that support the findings of this study are available from the corresponding author on request.

## Additional information

**How to cite this article:** França, G. S. *et al*. Host gene constraints and genomic context impact the expression and evolution of human microRNAs. *Nat. Commun.* 7:11438 doi: 10.1038/ncomms11438 (2016).

## Supplementary Material

Supplementary Information Supplementary Figures 1-11 and Supplementary References

Supplementary Data 1 Enriched biological processes in the set of host genes using AmiGO 2.

Supplementary Data 2 Enriched biological processes in the set of host genes using DAVID.
