# Supplementary material for: Host gene constraints and genomic context impact the expression and evolution of human microRNAs
Source: Nat Commun. 2016 Apr 25;7:11438. doi: 10.1038/ncomms11438 (PMC4848552; doi:10.1038/ncomms11438)
Supplement: Supplementary Information — Supplementary Figures 1-11 and Supplementary References [file ncomms11438-s1.pdf]

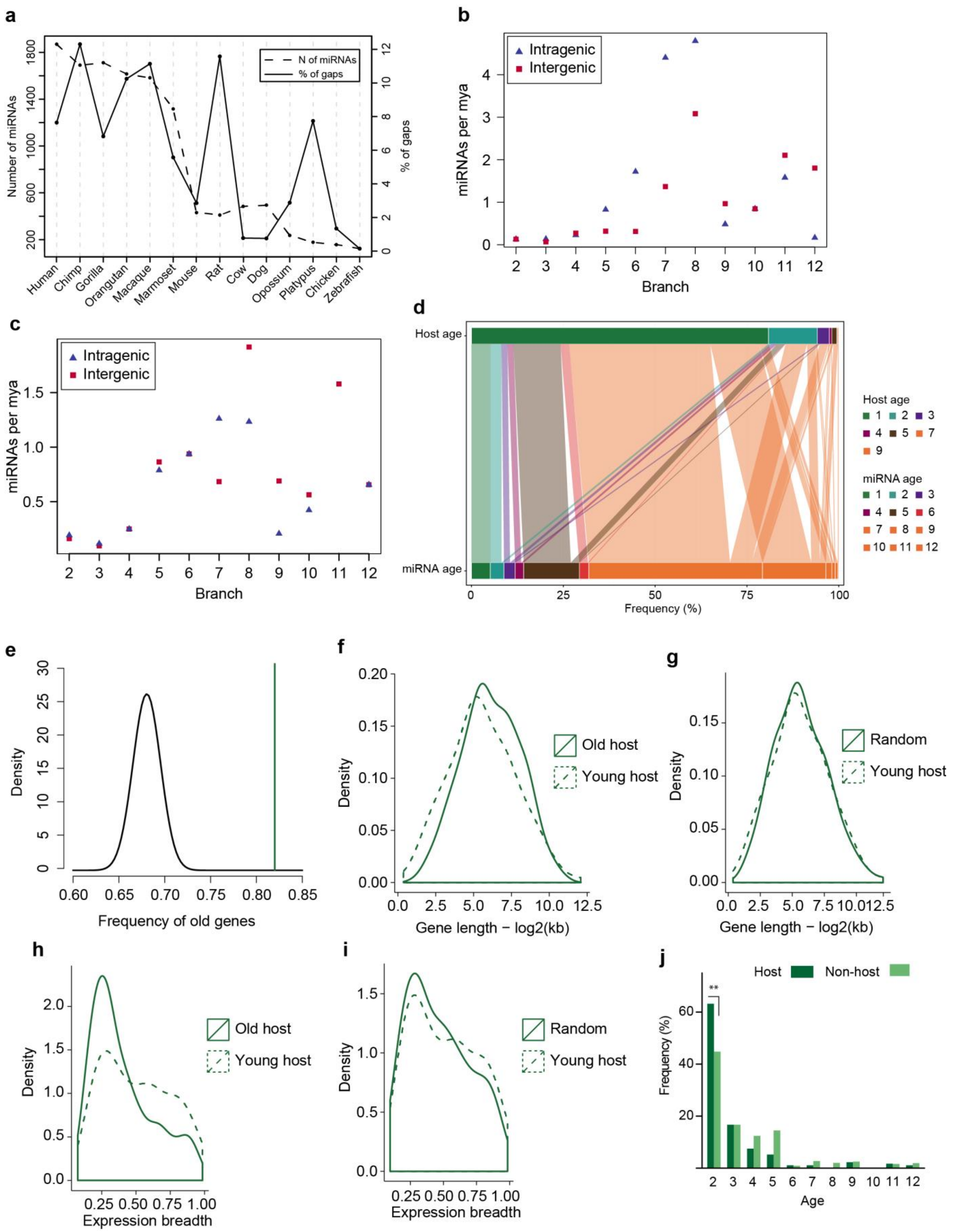
a
Number of miRNAs
% of gaps
N of miRNAs
% of gaps
Human
Chimp
Gorilla
Orangutan
Macaque
Marmoset
Mouse
Rat
Cow
Dog
Opossum
Platypus
Chicken
Zebrafish
b
Intragenic
Intergenic
miRNAs per mya
Branch
c
Intragenic
Intergenic
miRNAs per mya
Branch
d
Host age
miRNA age
Frequency (%)
Host age
miRNA age
e
Density
Frequency of old genes
f
Old host
Young host
Density
Gene length − log2(kb)
g
Random
Young host
Density
Gene length − log2(kb)
h
Old host
Young host
Density
Expression breadth
i
Random
Young host
Density
Expression breadth
j
Host
Non-host
**
Frequency (%)
Age

Supplementary Figure 1. Human inter- and intragenic miRNAs across the vertebrate lineage and age of host genes. (a) Relationship between the number of human miRNAs found in each branch and genome gap content (fraction of the genomes containing gapped regions). Genome quality did not undermined miRNA identification since close species have similar number of homologous miRNAs and discrepant gap content proportions (ex: chimp vs gorilla, mouse vs rat, platypus vs chicken, etc.). **(b)** Number of human inter- and intragenic miRNAs emerged in each branch across the vertebrate lineage. Here we analyzed only expressed miRNAs and those distant from 10 kb to each other were merged into a single cluster. The representative miRNA of the cluster was randomly picked. Number of miRNAs per million years (mya) were calculated by the ratio of the number of inter- or intragenic miRNAs emerged in each branch (see Fig. 1A, main text) to the time elapsed from the previous branch. For example, the gain rate of intergenic miRNAs in branch 2 (chicken) is $N_{inter} / D_{\mathrm{b12\text{-}b1}} - D_{\mathrm{b12\text{-}b2}}$, where N_{inter} is the number of intergenic miRNAs emerged in branch 2; $D_{\mathrm{b12\text{-}b1}}$ is the divergence time between branch 12 (human) and branch 1 (fish) and $D_{\mathrm{b12\text{-}b2}}$ is the divergence time between branch 12 and branch 2 (chicken). Significant differences (branches 5, 6, 7, 8, 12; $P < 0.05$) between the quantities of inter- and intragenic miRNAs in each branch were assessed by Binomial tests assuming equal probabilities (0.5). Divergence times were obtained from timetree.org. **(c)** Number of human inter- and intragenic miRNAs emerged in each branch across the vertebrate lineage. Here we analyzed only the miRNAs annotated by Fromm et al. (2015)[1]. This dataset consists of a re-annotation of miRBase (v.21) entries based on stringent criteria to exclude non-bona fide miRNAs. For humans, the number of true-positives was reduced to one third of the miRBase entries. Even with this highly stringent annotation, the excess of intragenic miRNAs in branch 7 persists ($P < 0.05$, binomial-test). The excess of inter- or intragenic miRNAs was not significant in all other branches. **(d)** Age relationships among intragenic miRNAs and their host genes. Horizontal line lengths are proportional to the frequency of miRNAs and host genes of each age. **(e)** Null distribution of the frequency of old genes. Equal-sized sets of genes just as or older than the sets of miRNAs owing a particular age were randomly sampled 10,000 times (SD = 0.015; 95% CI = 0.68 ± 0.0002). The observed greater proportion of old host genes is represented by the vertical line ($P < 0.0001$ or $P = 3.93 \times 10^{-11}$, chi-squared test). **(f)** Length distribution of host genes. Gene length was given by the distance between annotated start and end genomic coordinates represented in log2 scale. We used the longest gene transcript as the reference and single exon genes were excluded. Distributions show that old genes are longer than young ones ($P = 0.002$, Mann-Whitney U test). **(g)** Length distribution of host genes

controlling for young host length. We generated a random distribution centered on the approximate median of young host lengths to rule out a possible bias as old genes are longer than young ones. Adopting this procedure, no significant difference between the two distributions was observed ($P = 0.78$, Mann-Whitney U test) but we still observed a similar proportion (80%) of old host genes as the original data (*i.e.* no difference compared to the whole dataset: $P = 0.57$, chi-squared test). **(h)** Expression breadth[2] of old and young host genes. Old host genes are more broadly expressed (lower τ) than young ones ($P = 1.4 \times 10^{-6}$, Mann-Whitney U test). **(i)** Expression breadth distribution of host genes controlling for young host expression breadth. Similarly to Fig. S1G, we generated a random distribution centered on the approximate median of young host expression breadth to rule out a possible bias as old genes have higher expression breadth than young ones. Adopting this procedure, no significant difference between the two distributions was observed ($P = 0.2$, Mann-Whitney U test) but we still observed the similar proportion (81.8%) of old host genes as the original data (*i.e.* no difference compared to the whole dataset: $P = 0.76$, chi-squared test). **(j)** Young host and non-host genes' frequency according to their ages. Single exon genes were excluded to avoid new gene bias in non-host genes due to excess of retrogenes. Also for young genes (age ≥ 2) frequencies of each age are different between the two categories. Host genes are overrepresented for age 2 (the oldest one among young genes) ($P < 0.0001$, chi-squared test).

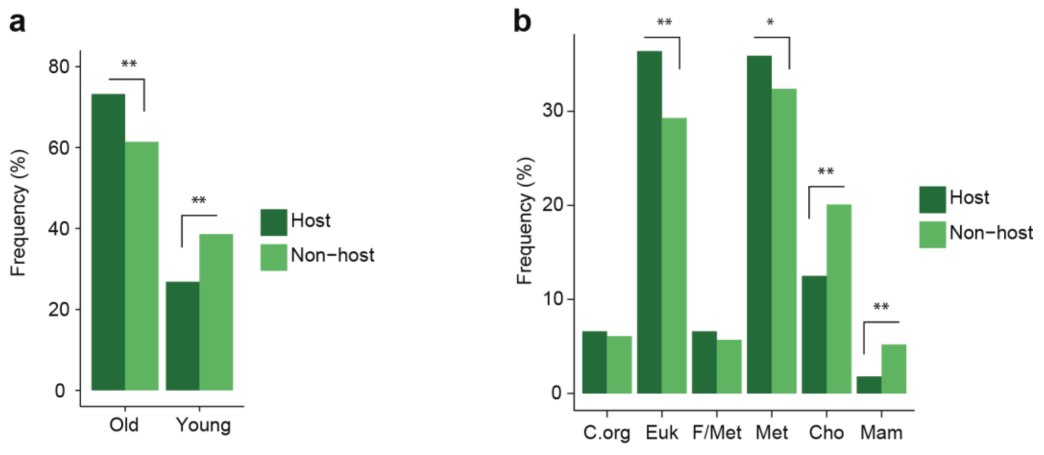

Supplementary Figure 2. Age of host and non-host genes using alternative dating methods. (a) Frequency of old and young host and non-host genes using Ensembl orthology (v.71). The 1:1 orthologs between human and additional 29 species were obtained from Ensembl (see Supplementary Fig. 11). Old genes were defined as those with orthologs in at least one fish species and young genes were those emerged after the fish divergence (Supplementary Fig. 11). A chi-squared test revealed that host genes

are overrepresented in the Old group relative to the Young ($P = 1.88 \times 10^{-13}$). **(b)** Host and non-host gene frequency according to age classes defined by Chen et al. (2012)[3]. Their dataset provides ages for mouse genes defined as follows: Cellular organisms (C. org); Eukaryota (Euk); Fungi/Metazoa (F/Met); Metazoa (Met), Chordata (Cho) and Mammalia (Mam). We then used the human-mouse 1:1 orthologs obtained from Ensembl to assign these age classes to host and non-host genes. Using chi-squared tests, we observed that host genes are overrepresented (in respect to non-hosts) in older groups (Eukaryota: $P = 4.0 \times 10^{-5}$ and Metazoa $P = 0.05$), whereas are underrepresented in younger groups (Chordata: $P = 8.79 \times 10^{-9}$ and Mammalia: $P = 4.4 \times 10^{-7}$).

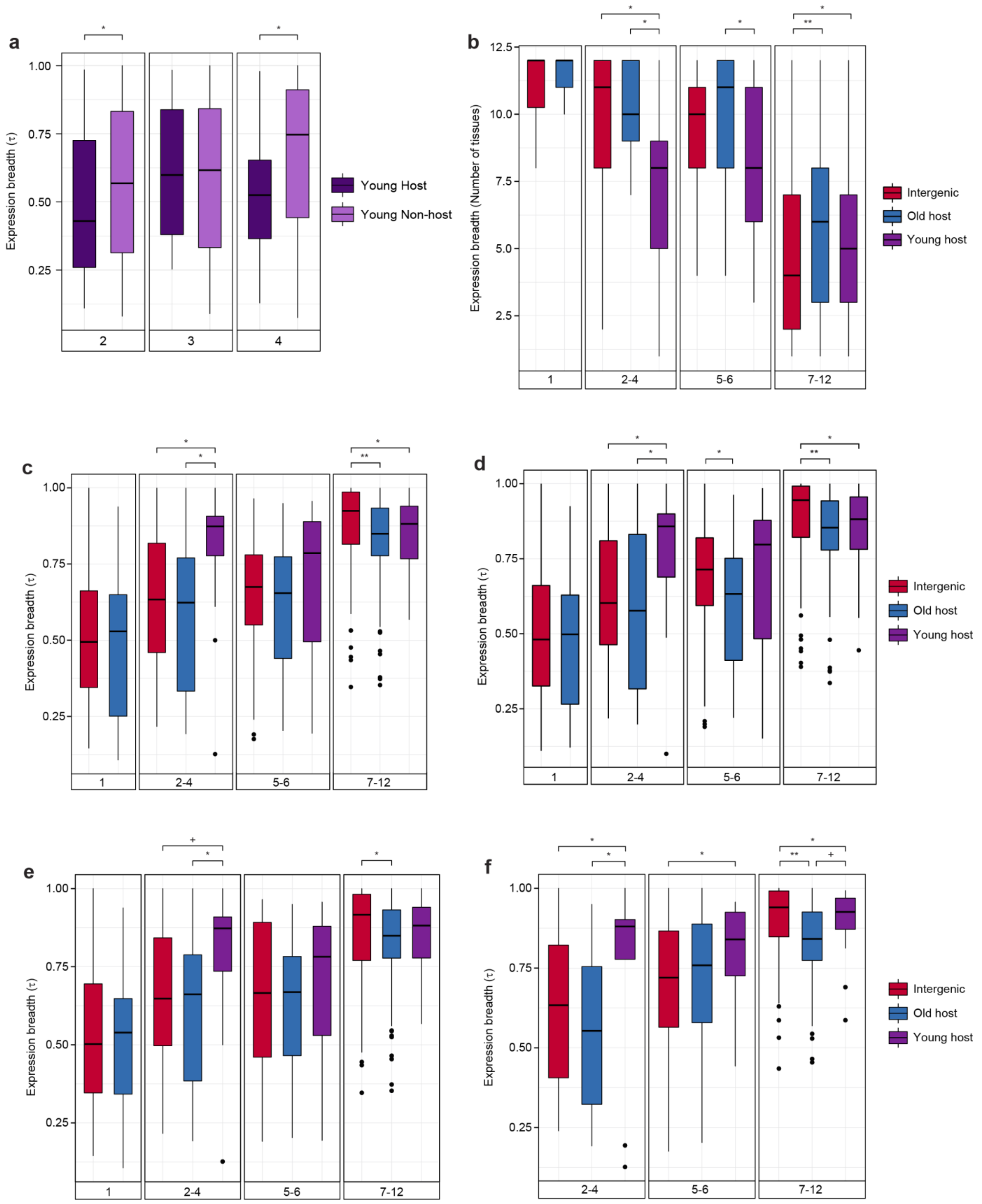
a
b
c
d
e
f
Expression breadth (τ)
Expression breadth (Number of tissues)
Young Host
Young Non-host
Intergenic
Old host
Young host
1.00
0.75
0.50
0.25
12.5
10.0
7.5
5.0
2.5
1
2
3
4
2-4
5-6
7-12
*
**
+

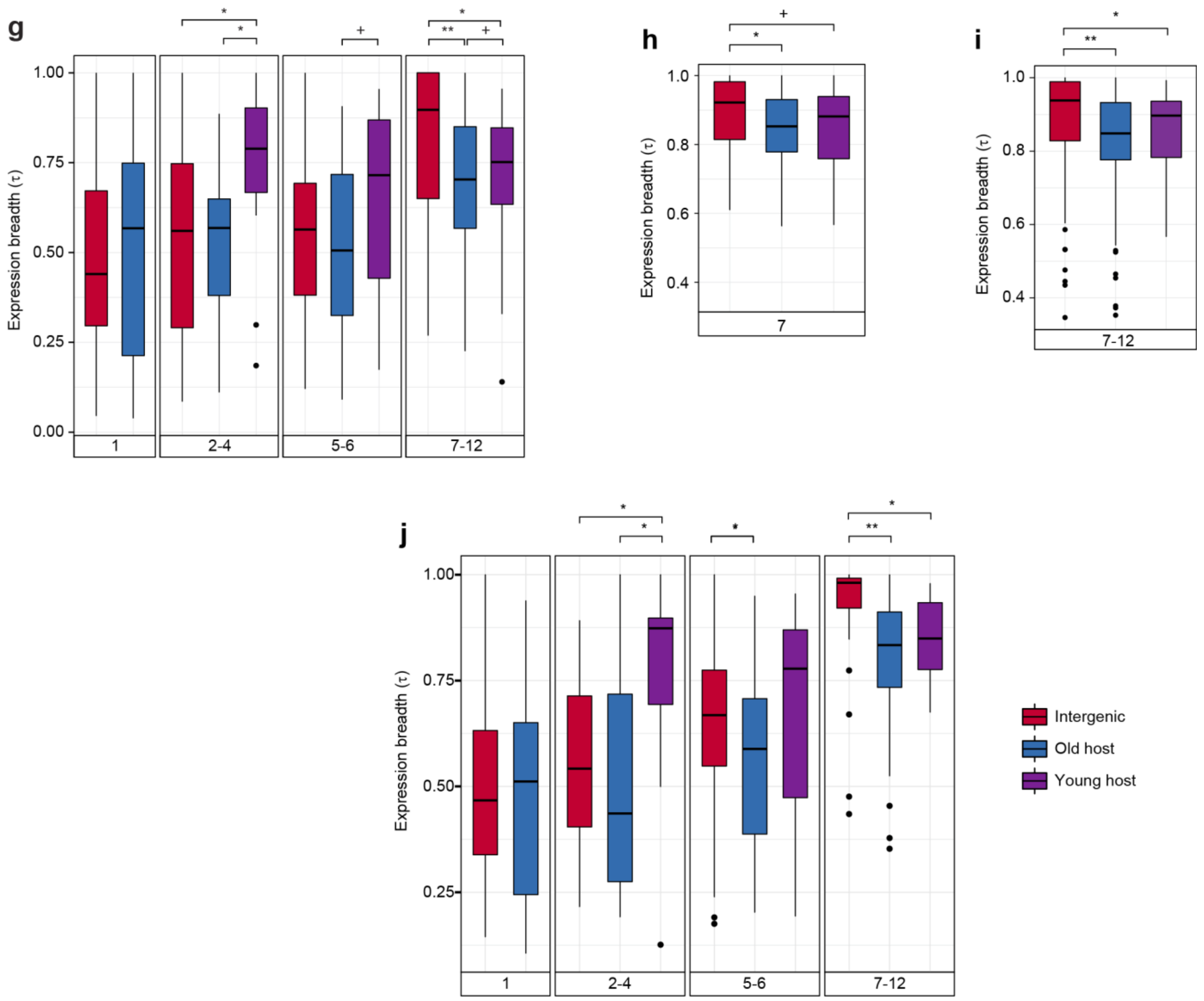

Supplementary Figure 3. Human miRNA expression breadth according to their age and genomic location. (a) Expression breadth of young host and non-host genes. Young hosts are more broadly expressed than young non-host genes of age 2 (P = 0.007, Mann-Whitney U test) and of age 4 (P = 0.001). **(b)** Expression breadth of miRNAs determined by the number of tissues in which they are expressed. **(c)** Expression breadth excluding expression from testis. **(d)** Expression breadth excluding expression from brain and cerebellum. **(e)** Expression breadth of miRNAs using the merged dataset of those distant 10 kb from each other. **(f)** Expression breadth of miRNAs using ages defined by Iwama et al. (2012)[4]. **(g)** Expression breadth using RNA-seq data obtained from the same study (Meunier et al. 2013)[5], measured for five tissues (brain, cerebellum, heart, kidney and testis). **(h)** Expression breadth of young miRNAs only including those of age 7. **(i)** Expression breadth excluding canonical, 5-tailed and 3-tailed mirtrons.

As 94% of the annotated mirtrons in our dataset emerged after the rodent-primate split, only miRNAs of age class 7-12 were considered. **(j)** Expression breadth of miRNAs annotated by Fromm et al. (2015)[1], which is a highly curated dataset of bona-fide miRNAs from miRBase entries. Even for this restricted but robust dataset the same patterns relating miRNA expression breadth and the age of their host genes could be observed, notably for young miRNAs (7-12) within old host genes. Boxplots in red represent intergenic miRNAs, blue are intragenic miRNAs within old host genes and purple are intragenic miRNAs within young host genes. (+) $0.05 < P < 0.09$; (*) $P < 0.05$; (**) $P < 0.001$. Statistical differences were assessed by Mann-Whitney U tests. An expression threshold of 1 cpm (counts per million) in at least one tissue/sample was considered.

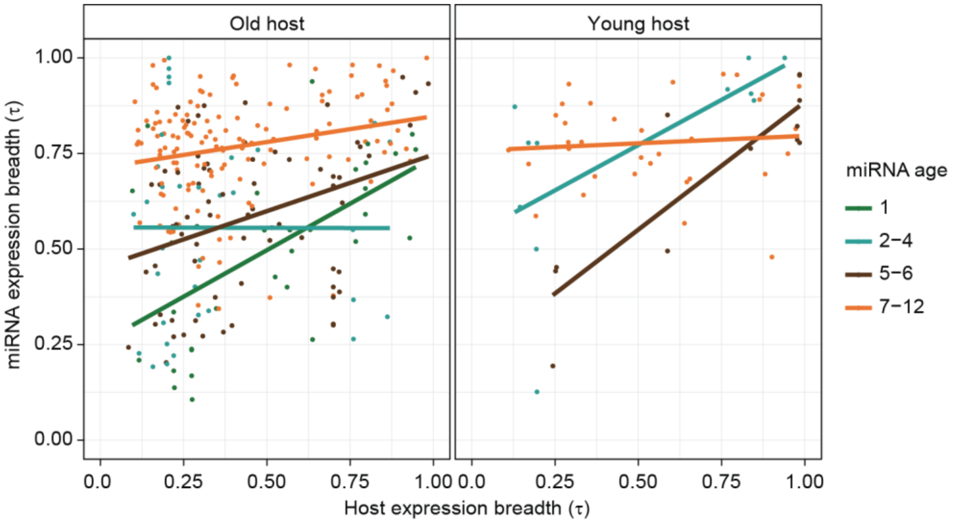

Supplementary Figure 4. Expression breadth correlations between intragenic miRNAs and their host genes. Expression breadth correlations between intragenic miRNAs and their host genes, regarding miRNA age classes and of their host genes. Age 1 in old hosts: $\rho = 0.55$, $P = 0.001$; Age 2-4: in old hosts: $\rho = 0.12$, $P = 0.49$, in young hosts: $\rho = 0.66$, $P = 0.02$; Age 5-6: in old hosts: $\rho = 0.34$, $P = 0.004$, in young hosts: $\rho = 0.84$, $P = 0.0006$; Age 7-12: in old hosts: $\rho = 0.17$, $P = 0.04$, in young hosts: $\rho = 0.19$, $P = 0.57$. The expression breadth of miRNAs and host genes were determined using the tissue-specificity index $(\tau)^2$ using 12 and 16 tissues, respectively (see Methods in the main text).

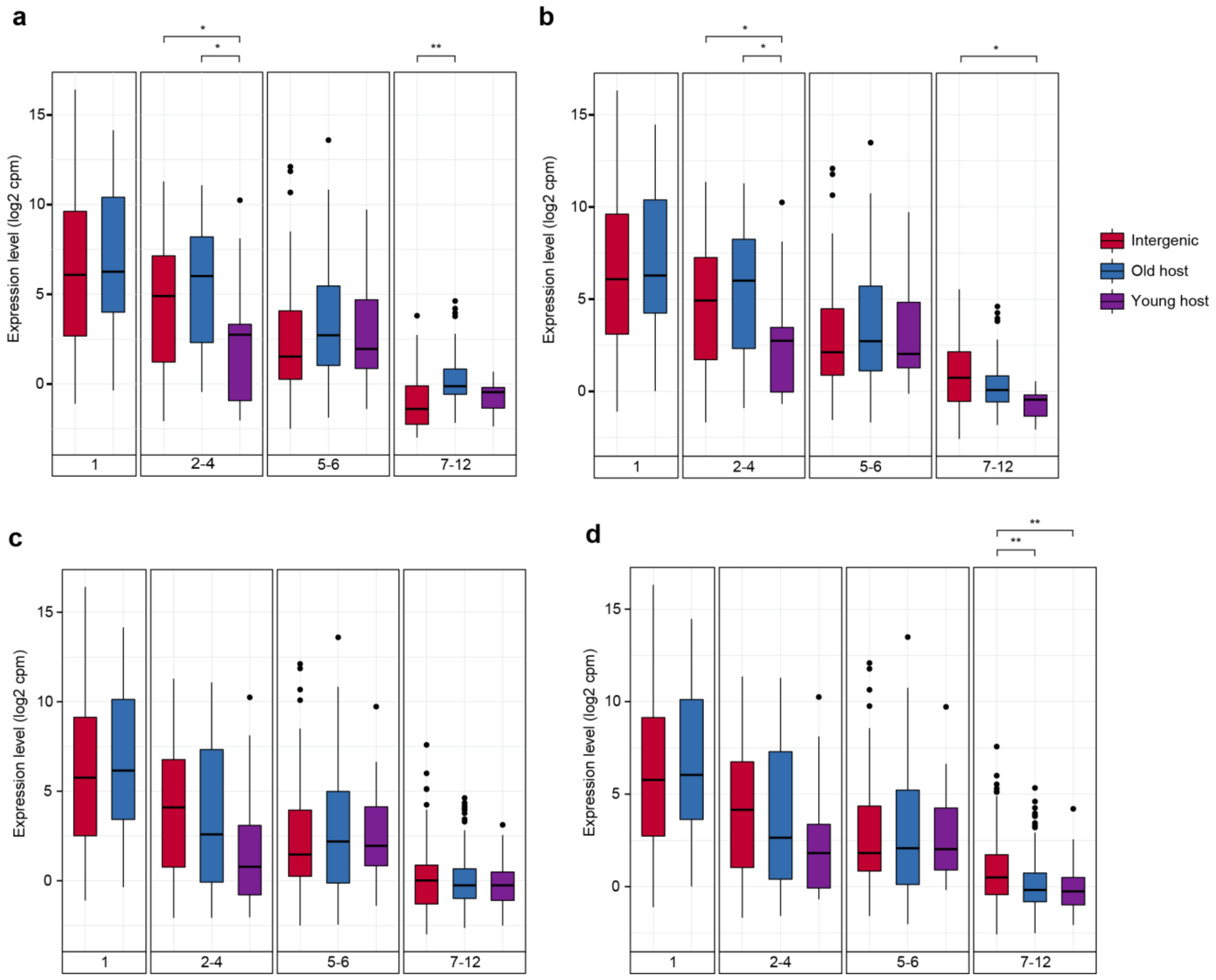

Supplementary Figure 5. Human miRNA expression levels according to their age and genomic context. (a) Boxplots of the median of miRNA expression levels based on miRNAs annotated by Fromm et al. (2015)[1] (www.mirgenedb.org). The median was calculated considering the tissues in which the miRNA is expressed (> 1cpm). Red boxplots represent intergenic miRNAs, blue are the intragenic miRNAs within old hosts and purple are intragenic within young hosts. It is noticeable that expression levels increase with miRNA age (i.e. older). Interestingly, by excluding expression from testis, young intragenic miRNAs (age 7-12) within old host genes are more highly expressed than the intergenic counterpart. **(b)** When we considered all 12 tissues (i.e., including testis) and MirGeneDB annotation, the difference in A was not observed, indicating testis-specific expression for young intergenic miRNAs.

(c) Boxplots of the median of miRNA expression levels based on the miRNAs annotated by miRBase. By excluding expression from testis, no significant differences were observed between miRNAs of the same age classes. **(d)** When we considered all 12 tissues (i.e., including testis) and miRBase annotation we observed that young intergenic miRNAs (age 7-12) are more highly expressed than young intragenic miRNAs. The difference in C is therefore likely due to testis-specific expression. (** $P < 0.0001$; * $P < 0.06$; Mann-Whitney *U* test).

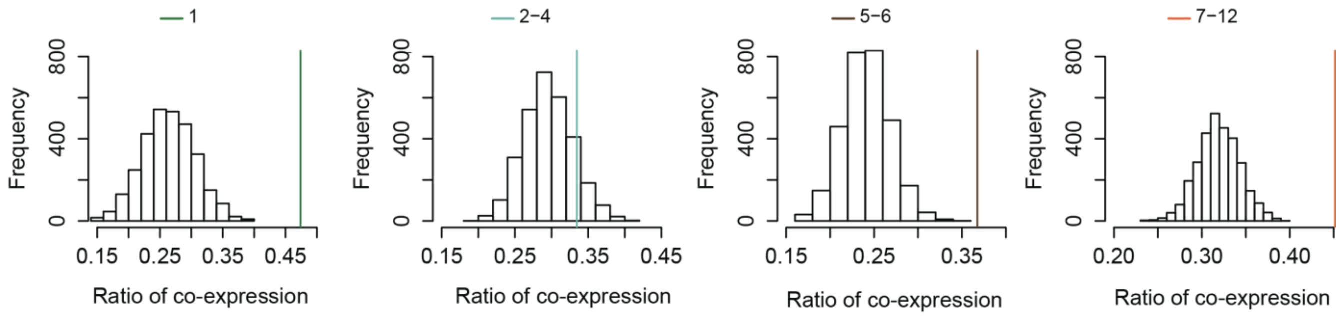

Supplementary Figure 6. Co-expression analysis of intragenic miRNAs and their host genes. Histograms represent the null distributions of the average of co-expression ratios between miRNAs and their host genes. We used expression datasets from Meunier et al. (2013)[5] and Brawand et al. (2011)[6] to assess miRNA and host gene expression for the same tissues (brain, cerebellum, heart, kidney and testis). For each miRNA-host pair, we ranked the tissues by expression levels and determined the ratio of the number of tissues in which miRNA and host genes were expressed in the same rank order. The average of observed co-expression ratios (represented by vertical lines) was compared with the averages of co-expression ratios by randomly shuffling the tissue rank order 3,000 times. Age 1: $P < 0.001$; Age 2-4: $P = 0.12$; Age 5-6: $P < 0.001$; Age 7-12: $P < 0.001$.

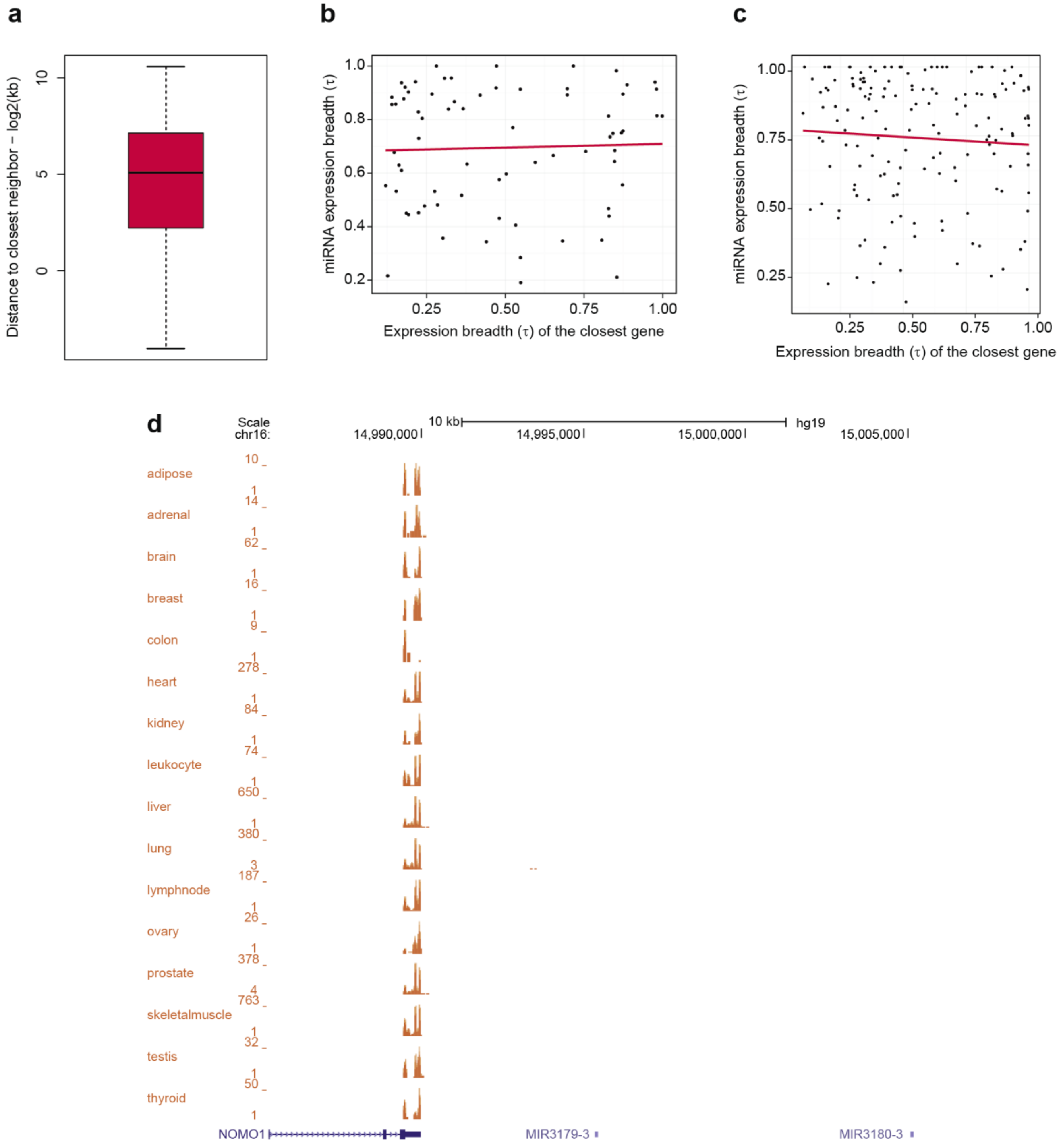

Supplementary Figure 7. Intergenic miRNAs and genomic context. (a) Boxplot of the distance between intergenic miRNAs and their closest coding genes. The y-axis is in log2 scale of the distance in kb (kilobases). The median is 33 kb. **(b)** Expression breadth correlation between intergenic miRNAs and their closest neighbors considering miRNAs distant up to 10 kb from their closest genes. No significant

correlation was found. **(c)** Expression breadth correlation between intergenic miRNAs and their closest neighbors considering the closest genes as those in the sense orientation and downstream to the miRNAs (i.e. miRNAs upstream to the closest gene). No significant correlation was found. **(d)** Example of reads spanning the 3' end of the closest gene (NOMO1) and proximal miRNAs (mir-3179-3 and mir-3180-3). To rule out the possibility that the lack of correlations in B and C were due to possible extended transcription of closest genes and proximal miRNAs in a tissue-specific manner, we sought for reads spanning the interval of gene 3'end and proximal miRNAs in 16 tissues (Illumina Human Body Map 2.0). No evidence of this mechanism was found. A similar pattern was observed for all 20 miRNAs downstream to the closest gene up to 10 kb.

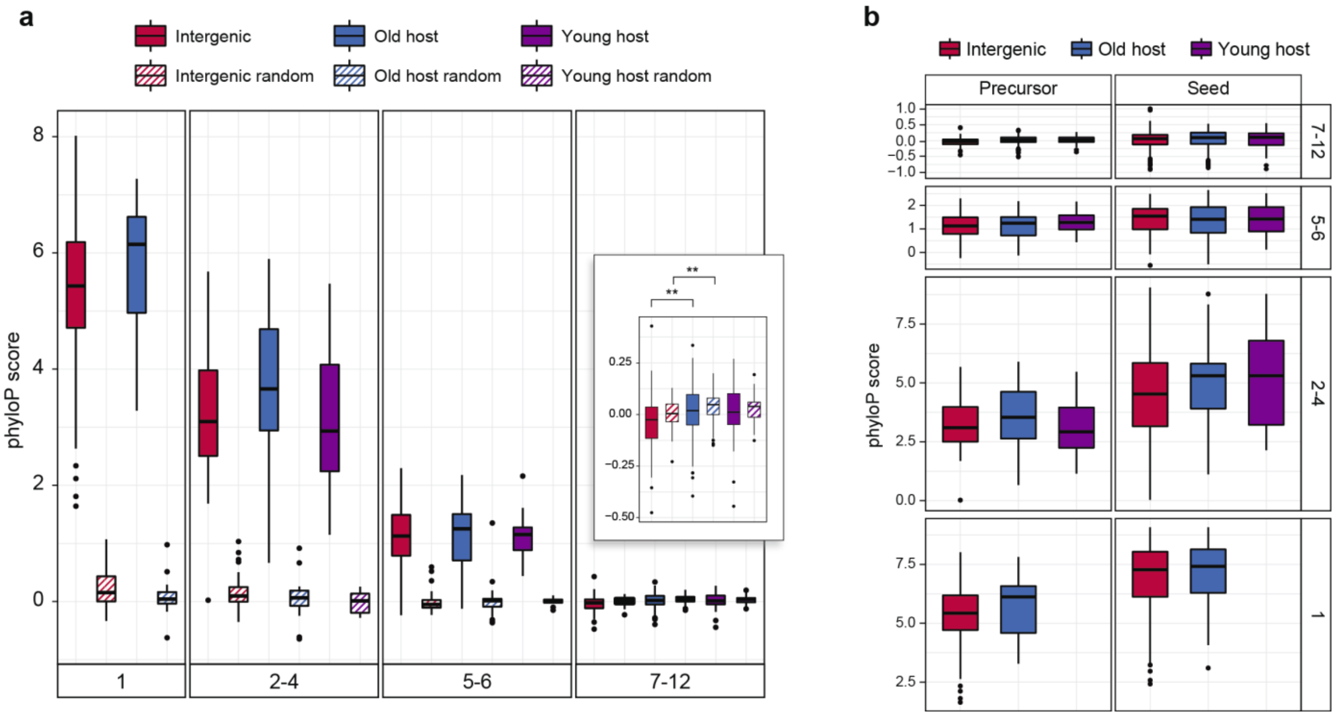

Supplementary Figure 8. Sequence conservation of human miRNAs. (a) Sequence conservation is represented by the average of phyloP scores along the precursor sequence. It is noticeable that older miRNAs have higher phyloP scores, reflecting our age class definitions. We detected a significant score difference between young (age 7-12) intragenic miRNAs within old host genes (blue) and young intergenic (red) miRNAs (**, $P < 0.001$, Mann-Whitney U test). However, we noticed that this difference also appear when we compare the random backgrounds (see Methods in the main text). Thus, the mentioned difference is likely due to the surrounding genomic region, rather than a differential

conservation of the precursor sequences. **(b)** Conservation of the seed sequences compared to the precursors. All comparisons in the same age classes (and also with the random background) revealed a significant higher conservation of the seed sequence (all $P < 0.01$, Mann-Whitney U test), except for intragenic miRNAs of ages 5-6 and 7-12 within young hosts. Taking into account the relatively short evolutionary time since the divergence of primates, it is notable that the seed of young miRNAs exhibited higher scores in respect to both the precursor and random background. However, although this could suggest a prompt seed conservation, a careful interpretation is needed because in addition to the methodological difficulty to detect selection over such a brief time scale, their phyloP scores are actually on the range of the neutral expectation (close to 0.0).

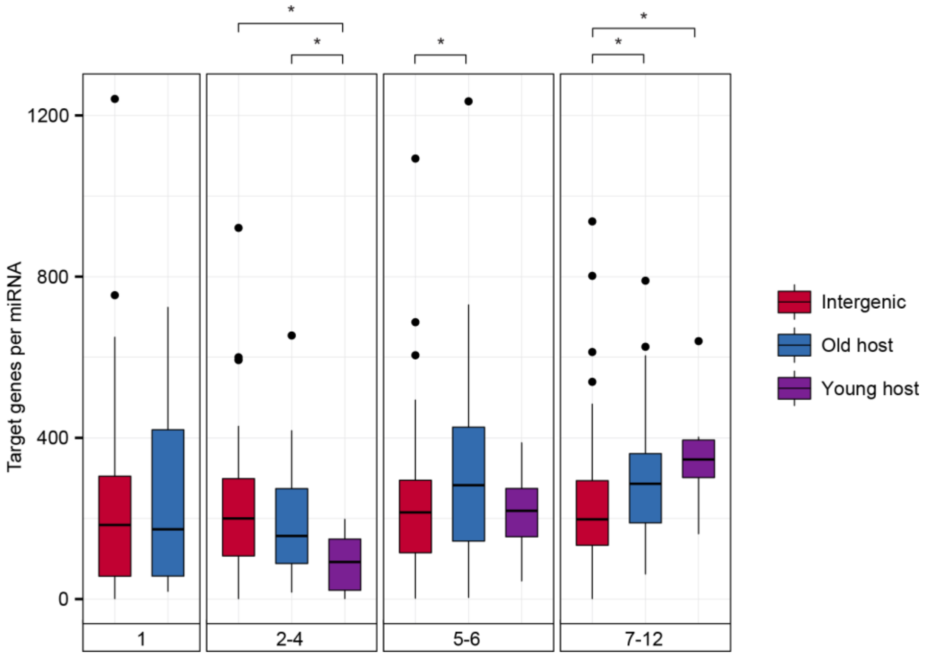

Supplementary Figure 9. Number of predicted target genes per miRNA using a stringent miRNA annotation. Boxplots for the distributions of the number of target genes per miRNA predicted using TargetScan 7 algorithm. Significant differences were assessed by Mann-Whitney tests (* $P < 0.05$). Here we used the stringent set of miRNAs annotated by Fromm et al. (2015)[1] to exclude potential non-bona fide miRNAs.

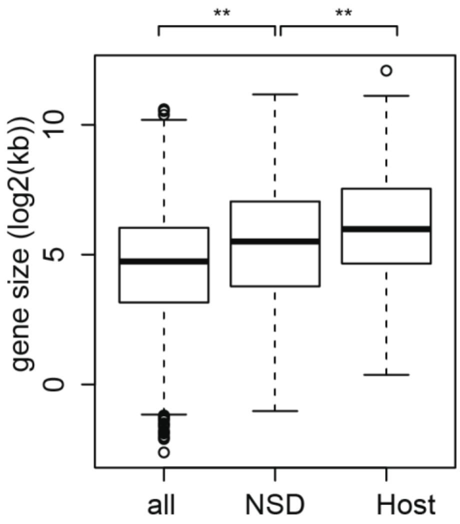

Supplementary Figure 10. Gene size of nervous system development genes. All genes annotated as involved in nervous system development (NSD) (GO:0007399) tend to be longer than all other protein-coding genes and shorter than host genes. To evaluate whether the enrichment of host genes associated with neural functions (Fig. 5B, manuscript) is related to gene size, we randomly sampled genes with similar size of NSD genes and performed a GO enrichment analysis with DAVID 6.7. None of the 100 random samples was enriched for neural-associated terms, suggesting that the enrichment of host genes for neural functions is not biased towards gene size (**) $P < 0.0001$.

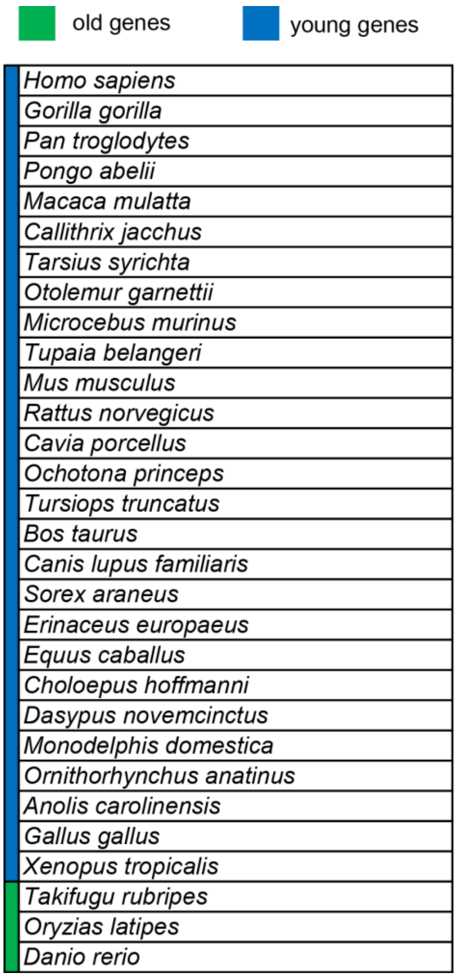

Supplementary Figure 11. List of species used to define gene ages by Ensembl orthology. Sets of 1:1 orthologs among human and other 29 species were obtained from Ensembl (v. 71) to define old and young genes. Old genes were defined as those with orthologs found in fishes and young genes were found in species emerged after fish divergence.

Supplementary References

1. Fromm, B. *et al.* A Uniform System for the Annotation of Vertebrate microRNA Genes and the Evolution of the Human microRNAome. *Annu. Rev. Genet.* **49,** 213-242 (2015).
2. Yanai, I. *et al.* Genome-wide midrange transcription profiles reveal expression level relationships in human tissue specification. *Bioinformatics* **21,** 650–659 (2005).
3. Chen, W.-H., Trachana, K., Lercher, M. J. & Bork, P. Younger genes are less likely to be essential than older genes, and duplicates are less likely to be essential than singletons of the same age. *Mol. Biol. Evol.* **29,** 1703–1706 (2012).
4. Iwama, H., Kato, K., Imachi, H., Murao, K. & Masaki, T. Human microRNAs originated from

two periods at accelerated rates in mammalian evolution. *Mol. Biol. Evol.* **30,** 613–626 (2013).

5. Meunier, J. *et al.* Birth and expression evolution of mammalian microRNA genes. *Genome Res.* **23,** 34–45 (2013).

6. Brawand, D. *et al.* The evolution of gene expression levels in mammalian organs. *Nature* **478,** 343–348 (2011).
